# Benthic habitat is an integral part of freshwater *Mysis* ecology

**DOI:** 10.1111/fwb.13594

**Published:** 2020-07-23

**Authors:** Jason D. Stockwell, Brian P. O’Malley, Sture Hansson, Rosaura J. Chapina, Lars G. Rudstam, Brian C. Weidel

**Affiliations:** ^1^ Rubenstein Ecosystem Science Laboratory University of Vermont Burlington VT U.S.A.; ^2^ U.S. Geological Survey Great Lakes Science Center Oswego NY U.S.A.; ^3^ Department of Ecology, Environment, and Plant Sciences Stockholm University Stockholm Sweden; ^4^ Department of Natural Resources Cornell University Ithaca NY U.S.A.

**Keywords:** detritus, diel vertical migration, mysids, omnivore, predation risk

## Abstract

1. Diel vertical migration (DVM) is common in aquatic organisms. The trade‐off between reduced predation risk in deeper, darker waters during the day and increased foraging opportunities closer to the surface at night is a leading hypothesis for DVM behaviour.
2. Diel vertical migration behaviour has dominated research and assessment frameworks for *Mysis*, an omnivorous mid‐trophic level macroinvertebrate that exhibits strong DVM between benthic and pelagic habitats and plays key roles in many deep lake ecosystems. However, some historical literature and more recent evidence indicate that mysids also remain on the bottom at night, counter to expectations of DVM.
3. We surveyed the freshwater *Mysis* literature using Web of Science (WoS; 1945–2019) to quantify the frequency of studies on demographics, diets, and feeding experiments that considered, assessed, or included *Mysis* that did not migrate vertically but remained in benthic habitats. We supplemented our WoS survey with literature searches for relevant papers published prior to 1945, journal articles and theses not listed in WoS, and additional references known to the authors but missing from WoS (e.g. only 47% of the papers used to evaluate in situ diets were identified by WoS).
4. Results from the survey suggest that relatively little attention has been paid to the benthic components of *Mysis* ecology. Moreover, the literature suggests that reliance on *Mysis* sampling protocols using pelagic gear at night provides an incomplete picture of *Mysis* populations and their role in ecosystem structure and function.
5. We summarise current knowledge of *Mysis* DVM and provide an expanded framework that more fully considers the role of benthic habitat. Acknowledging benthic habitat as an integral part of *Mysis* ecology will enable research to better understand the role of *Mysis* in food web processes.

## 1 INTRODUCTION

In many deep freshwater systems, macrocrustaceans of the genus *Mysis* play important roles in the food web (Devlin et al., 2016; Lasenby, Northcote, & Furst, 1986; Sierszen et al., 2014). *Mysis* spp. are omnivorous (Grossnickle, 1982) and can feed on detritus, phytoplankton, benthic invertebrates, zooplankton, and even fish embryos (Seale & Binkowski, 1988). *Mysis* can alter ecosystem structure and function through intense predation on zooplankton, which results in competition with planktivorous fishes (Ellis et al., 2011; Goldman, Morgan, Threlkeld, & Angeli, 1979; Martinez & Bergersen, 1991). In addition to their competitive roles in food webs, *Mysis* also serve as important prey for both benthic and pelagic prey fishes and juvenile life‐stages of many piscivorous fish species (Elrod & O’Gorman, 1991; Gamble, Hrabik, Stockwell, & Yule, 2011; Gamble, Hrabik, Yule, & Stockwell, 2011).

*Mysis* exhibit diel vertical migration (DVM), whereby individuals ascend to the pelagic zone as light levels decline at sunset, and then descend to the bottom as sunrise approaches (Beeton & Bowers, 1982). Temperature and light gradients define pelagic conditions that limit the vertical extent of *Mysis* migration (Boscarino, Rudstam, Minson, & Freund, 2010; Boscarino, Rudstam, Tirabassi, Janssen, & Loew, 2010; Gal, Rudstam, & Johannsson, 2004; Rudstam, Danielsson, Hansson, & Johansson, 1989; Teraguchi, Hasler, & Beeton, 1975). *Mysis* assessments and specimen collections are often conducted at night, when *Mysis* can be sampled with relative ease using pelagic nets and hydroacoustics (Jude et al., 2018; McCoy, 2015; Watkins et al., 2015). For example, standard sampling protocols for *Mysis* in the Laurentian Great Lakes call for pelagic sampling to commence 1 hr post‐sunset when the population is assumed to have migrated (EPA, 2015). Such sampling strategies are based on the assumption that all or most of the population regularly migrates to the pelagic zone at night and that resultant samples quantitatively represent the population.

Recent studies, however, indicate that *Mysis* exhibit partial DVM, whereby a portion of the population does not ascend but remains on bottom at night (Euclide, Hansson, & Stockwell, 2017; Ogonowski, Duberg, Hansson, & Gorokhova, 2013; O'Malley, Dillon, Paddock, Hansson, & Stockwell, 2018; O'Malley, Hansson, & Stockwell, 2018). Although partial DVM in *Mysis* has been observed in a number of systems including the Laurentian Great Lakes (Bowers, 1988; Johannsson, Rudstam, Gal, & Mills, 2003) and the Baltic Sea (Rudstam et al., 1989), the magnitude of the population that remains benthic at night is not well understood and this portion of the population is typically excluded from population assessments and evaluations of the importance of *Mysis* in the ecosystem. We surveyed the literature to evaluate the extent to which benthic *Mysis* have been included in research. We restricted our analyses to freshwater *Mysis* (Porter, Meland, & Price, 2008) except for the marine *Mysis mixta*, as this species has been extensively studied in the Baltic Sea where it coexists with freshwater congenerics *Mysis relicta* and *Mysis salemaai* and has similar ecological roles (Salemaa, Tyystjarvimuuronen, & Aro, 1986; Salemaa, Vuorinen, & Välipakka, 1990). Our survey results suggest that benthic habitat is more important to *Mysis* ecology than previously assumed, and is necessary to consider this to better understand *Mysis*’ production and its roles in food webs. We conclude with a set of questions and hypotheses to help frame research directions to fill in knowledge gaps in *Mysis* ecology.

## 2 LITERATURE SURVEY ON *MYSIS* ECOLOGY

We conducted literature surveys for three aspects of freshwater *Mysis* ecology—demographics, in situ diets, and experiments that examined *Mysis* feeding behaviour. Each survey was initiated using Web of Science (WoS) with varying sets of search terms, spanning 1945–2019. For demographics, we surveyed WoS using the terms “*Mysis*” AND “abundance OR densit* OR biomass* OR growth OR size OR length OR distribution” under the field “Topic”. We used the terms “*Mysis*” AND “diet OR gut OR stomach” under the field “Topic” to survey WoS for in situ diets. For experiments on *Mysis* feeding behaviour, we used two sets of terms under the field “Topic”. The first included “Mysis” AND “feeding OR predation” AND “experiment” under the field “Topic”, and the second included “Mysis” AND “contaminant* OR toxi*” under the field “Topic”.

We examined the resultant titles and abstracts for relevance to each topic, and extracted the relevant information from each study. However, several relevant papers were not identified by the WoS search. For example, at the conclusion of the literature survey, only 47% of the papers used to evaluate in situ diets were identified by WoS. Therefore, during the survey, we examined the cited references in all relevant papers that were published prior to 1945 (the earliest year for WoS search), journal articles and theses that were not listed in WoS, and additional references known to the authors. We did not include papers that developed new sampling devices (Lasenby & Sherman, 1991) or relied solely on acoustics without net samples (Levy, 1991; Miller, 2003). Papers that relied on previously published values (Morgan, 1985; Morgan, Threlkeld, & Goldman, 1978; Sell, 1982; Sullivan & Rudstam, 2016) were not included. Theses and dissertations were included unless data were published in peer‐reviewed literature. In those cases (e.g. Morgan, 1985), information from the published papers was used. Studies that reported metrics but not methods were excluded (Adare & Lasenby, 1994; Lasenby & Langford, 1972). More details are provided in Tables S1–S3, and the results are summarised below. Also note that *Mysis* taxonomy was revised in the mid‐2000s, with new names given to four sibling species formerly known as *M. relicta* (*M*. *salemaai*, *Mysis* *segerstraeli*, *Mysis* *diluviana*, and *M*. *relicta*; Audzijonytė & Väinölä, 2005). With the exception of the high Arctic, the only mysid species in North America is *M. diluviana*. Even so, wherever we report a particular species, we refer to the name used in the cited publication.

**FIGURE 1:**
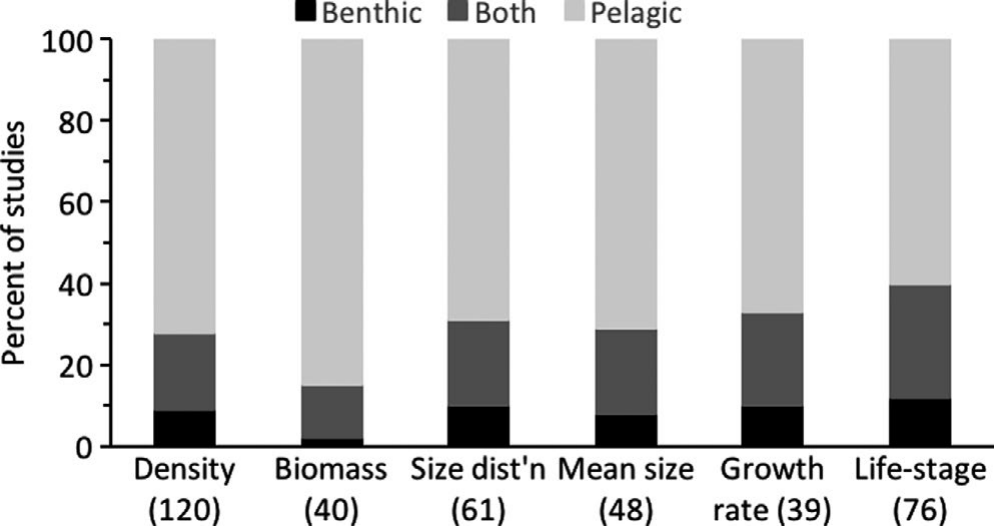
Percent of studies that reported freshwater *Mysis* spp. and *Mysis mixta* demographic data using individuals collected from benthic, pelagic, or both habitats. Number of studies reporting each type of demographic data is in parentheses. Total number of studies with at least one demographic metric was 142. Density (number of individuals per unit volume or area) includes abundance (total individuals)

**FIGURE 2:**
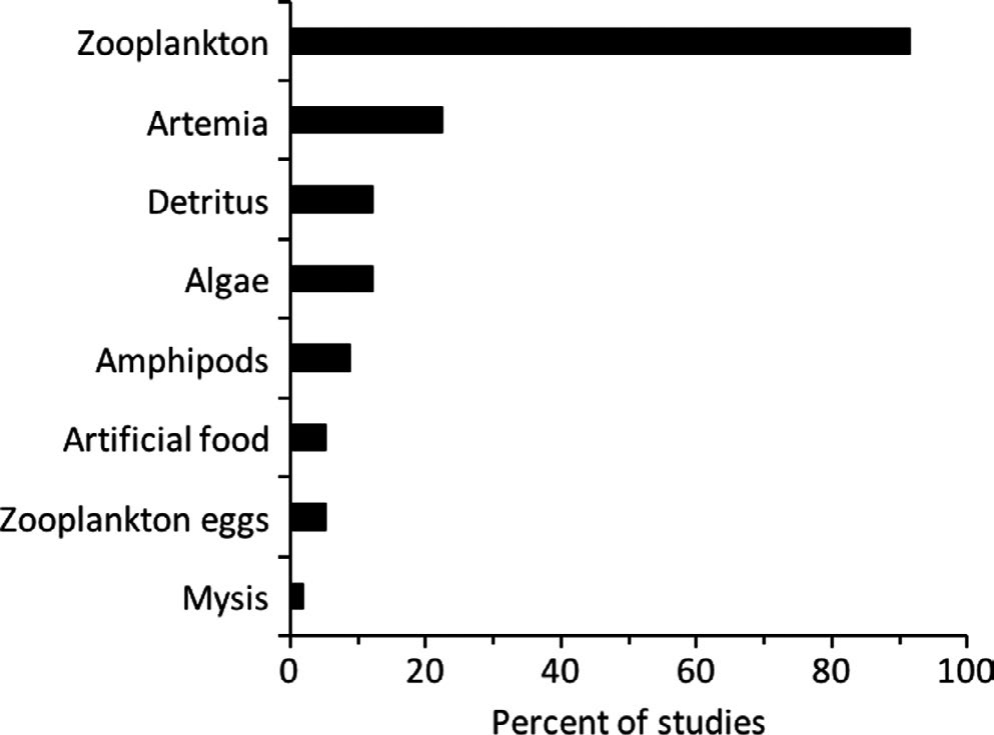
Different prey types used in freshwater *Mysis* spp. or *Mysis mixta* feeding experiments, presented as percent of studies (out of 58) that used each prey type. In some cases, multiple prey types were used in individual studies so sum of percentages exceeds 100%

The results of the literature survey clearly show that freshwater *Mysis* research has largely ignored benthic‐caught individuals and their benthic environment (Figures 1 and 2). *Mysis* demographics (*n* = 142 total studies) included benthic‐caught individuals in a minority of studies, ranging from 15% of the studies that estimated biomass (*n* = 40) to 40% of studies that assessed life‐stages (*n* = 76; Figure 1; Table S1). Conversely, such estimates were based solely on pelagic‐caught individuals in 60–85% of demographic studies. However, in a handful of studies which sampled pelagic and benthic habitats at night, variable but potentially very high proportions of *Mysis* have been reported on the bottom at night (3–84%, Table 1).

**TABLE 1:** Compilation of studies documenting partial diel vertical migration in *Mysis* spp. (*Mysis diluviana*,* Mysis relicta*,* Mysis mixta*,* Mysis salameii*) at night. Studies that did not report quantitative values of *Mysis* spp. on bottom at night were only included if they reported presence/absence

Species	Water body	Depth (m)	% on bottom at night	Method	Source
*M. relicta*	Superior	250	50	Submarine; Net	Bowers (1988)
*M. relicta*	Snasavatnet	48	23–70	Sled; Net	Moen and Langeland (1989)
*M. relicta*	Jonsvatn	10–80	7−84^a^	Sled; Net	Naesje et al. (2003)
*M. mixta*	Baltic	28–40	30	Sled; Net	Rudstam et al., (1989)
*M. relicta*	Ontario	35–100	< 5	Sled; Net	Shea and Makarewicz (1989)
*M. relicta*	Breiter, Schmaler Luzin	14–40	Pr	Scuba; Net	Waterstraat, Krappe, Riel, and Rumpf (2005)
*M. relicta*	Ontario	35–75	Pr	Predator diet	Brandt (1986)
*M. relicta*	Ontario	125	Pr	Sled; Net	Johannsson et al. (2001), Johannsson et al. (2003)
*M. mixta, M. relicta, M. salemaii*	Baltic	20–40	15	Camera	A. Staaf & S. Hansson (unpublished data, Stockholm Univ.)
*M. relicta, M. salemaii*	Baltic	30–35	Pr	Sled	Ogonowski et al. (2013)
*M. diluviana*	Champlain	70–120	Pr	Sled	Euclide et al. (2017)
*M. diluviana*	Champlain	60–100	3–46	Sled; Net	O'Malley, Hansson, et al. (2018)
*M. diluviana*	Champlain	60	Pr	Sled; Camera	O'Malley, Dillon, et al. (2018)

‘Pr’ indicates present. ‘Sled’ refers to benthic sled, sledge, or dredge; ‘Net’ refers to vertically or horizontally towed pelagic net.

^a^ Based on biomass.

Our survey of in situ diet studies revealed that benthic‐caught *Mysis* were examined in only 44% of identified studies (*n* = 32 total studies; Table S2), despite their well‐known benthic distributions and omnivorous diets, which can include detritus and benthic invertebrates (Johannsson et al., 2001, 2003; Lasenby & Shi, 2004; Lehtiniemi, Kiljunen, & Jones, 2009; Lehtiniemi, Viitasalo, & Kuosa, 2002; Parker, 1980). Pelagic‐caught individuals were solely examined in 56% of the identified in situ diet studies (Table S2). Detritus was reported in *Mysis* stomachs in 19 of the 32 studies, including nine of 10 studies that included both benthic‐ and pelagic‐caught *Mysis*, and somewhat surprisingly, in eight of 18 studies that only examined pelagic‐caught *Mysis*. If mysids feed continuously when on bottom, then pelagic‐caught *Mysis* sampled early in the night, soon after ascent, may still contain benthic resources in their guts. The remaining 10 pelagic‐only studies did not include detritus as a possible prey category (Table S2). In a recent study, benthic‐caught *Mysis* during the night and day had similar amounts of detritus in their stomachs, suggesting that *Mysis* feed continuously when on the bottom (O'Malley & Stockwell, 2019) and benthic resources are likely to contribute significantly to *Mysis* growth and production (Lehtiniemi et al., 2002; Sierszen, Kelly, Corry, Scharold, & Yurista, 2011; Whall & Lasenby, 2009).

We also found that *Mysis* feeding ecology experiments (*n* = 58 total studies) used benthic‐ or combined benthic‐ and pelagic‐caught individuals in only 21% of the studies, whereas 43% of the studies used only pelagic‐caught individuals and 36% did not report the habitat from which *Mysis* were captured for the experiments (Table S3). Pelagic zooplankton was by far the most used prey item in feeding experiments (>90% of experimental studies), followed by brine shrimp *Artemia*, which is not a natural prey of *Mysis* (22% of studies; Figure 2, Table S3). We found few studies where detritus, benthic invertebrates, or algae were used in experiments (*n* ≤ 7 each, Figure 2, Table S3) despite their presence in field diets (see *Role of benthic food resources* below).

The lack of historical focus on the benthic environment may bias our basic understanding of *Mysis* ecology and the role of *Mysis* in ecosystems. As evidenced by findings from studies that have examined benthic and pelagic *Mysis* at night (Table 1), only a fraction of a population may inhabit the pelagic environment at night (Naesje, 1995). The ecology of* Mysis* is more complex than that of an animal simply hiding on the bottom during the day waiting for the cover of darkness to migrate up the water column at night to feed on plankton.

Researchers have probably focused on the pelagic phase of *Mysis* for several reasons. First, *Mysis* DVM has been assumed to be a population‐level phenomenon and consequently, night pelagic samples should provide representative estimates of abundance and other population demographics. Second, pelagic habitat is easier to sample than benthic habitat. Sampling devices towed along the bottom can fill with substrate (e.g. mud, silt, detritus, dreissenid mussel shells), get caught on obstructions, or not hold tight to bottom with sharp bathymetric relief or complex substrate. Towing a net through the water column is far easier and safer, despite the necessity of working at night. A number of early studies concluded that pelagic vertical net tows at night were the best method for quantitative estimates of *Mysis* because they yielded the highest areal density estimates among methods (Grossnickle & Morgan, 1979; Morgan & Threlkeld, 1982; Nero & Davies, 1982; Sell, 1982; Shea & Makarewicz, 1989), which probably influenced following generations of *Mysis* researchers (but see Reynolds & DeGraeve, 1972). Third, *Mysis* introductions into non‐native lakes resulted in negative impacts on pelagic zooplankton in many cases (Goldman et al., 1979; Kinsten & Olsén, 1981; Lasenby et al., 1986), which may have increased effort to understand their pelagic implications for fisheries management at the expense of examining their benthic role. Similar consequences of *Mysis* introductions on benthic environments are not as apparent in the literature and probably more difficult to study and detect, although several studies have noted their importance as prey to benthic fish communities in systems affected by *Mysis* introductions (e.g. Ellis et al., 2011).

## 3 THE NEED FOR AN EXPANDED FRAMEWORK ON *MYSIS* BENTHIC RESOURCES AND HABITAT

The focus on pelagic *Mysis* restricts our understanding of its general ecology, and poses several potentially large knowledge gaps with implications for population assessments, food web ecology, and fisheries management (Kitchell et al., 2000; Pothoven & Madenjian, 2008; Pothoven, Nalepa, Schneeberger, & Brandt, 2001).

### 3.1 Mysis demographics and benthic habitat sampling

**FIGURE 3:**
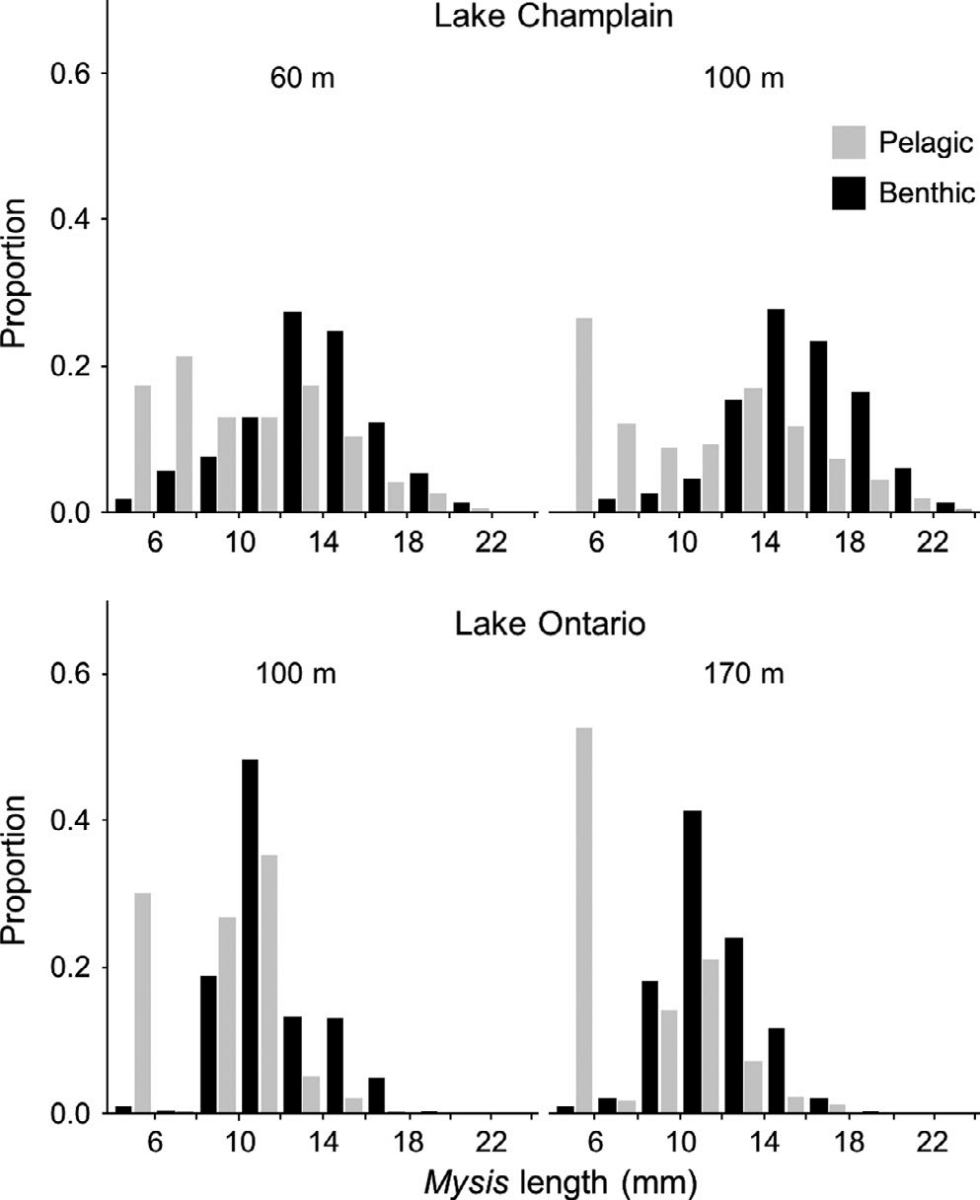
Length–frequency distributions of *Mysis diluviana* collected at night in pelagic and benthic habitats by site depth from Lake Champlain during June–November 2015 (O'Malley, Dillon, et al., 2018) and from Lake Ontario in May 2019 (Rosaura J. Chapina, unpublished data)

*Mysis* occupy benthic habitat night and day (Table 1). Evidence suggests that the individuals caught on the bottom both night and day are represented by disproportionately more adults, including gravid females, compared to pelagic individuals caught during the night (Figure 3; Euclide et al., 2017; McWilliam, 1970; O'Malley, Hansson, et al., 2018; Reynolds & DeGraeve, 1972). Consequently, assessments based on night pelagic samples may underestimate population density and biomass—the latter to a greater extent because of the exponential increase in mass with length. Population size–structure and life‐stages from night pelagic samples may also bias our inferences of population demographics.

Research using cameras (Bergersen & Maiolie, 1981) and video (O'Malley, Dillon, et al., 2018) indicate that benthic *Mysis* densities may be 2–10× greater than estimates based on traditional benthic sled collections, suggesting that estimates of the proportion of *Mysis* that are benthic at night based on benthic sleds (Table 1) are probably conservative. Further, wide variability in mysid density estimates has been observed in some benthic collections; however, where reported, the precision of benthic density estimates appears similar in magnitude to that of pelagic density estimates. For example, Moen and Langeland (1989) report coefficients of variation (CV) of 24 and 27% for day and night bottom samples collected with a beam trawl. Naesje, Saksgard, Jensen, and Sandlund (2003) found parallel (side‐by‐side) vertical water column hauls had an average CV of 27%, and among three stations vertical water column hauls had an average CV of 45%. Parallel bottom samples with a beam trawl had an average CV of 42%. O'Malley, Dillon, et al. (2018), O'Malley, Dillon, et al. (2018) found average CV of 23 (day) and 68% (night) for pelagic sampling, and 41 (day) and 32% (night) for benthic sampling. Understanding sampling gear bias and precision is critical for measuring potential demographic differences among habitats. Adoption and refinement of digital recording systems, in concert with benthic sleds, are promising methods to more fully assess mysids in benthic habitats. Deployment of such technologies can take a variety of forms, including stationary observatories (e.g. Grossman, Gallager, & Mitarai, 2014; O'Malley, Dillon, et al., 2018) and mobile platforms such as remotely operated vehicles, autonomous underwater vehicles, drop‐frames, benthic sleds, and fish trawls (e.g. Bethoney & Stokesbury, 2018; Brandt et al., 2013; Gutt & Siegel, 1994; Karatayev, Mehler, Burlakova, Hinchey, & Warren, 2018; Kilpatrick, Ewing, Lamb, Welsford, & Constable, 2011; Rosenkranz, Gallager, Shepard, & Blakeslee, 2008). Future methodological evaluations and sampling recommendations for benthic *Mysis* would be valuable and probably increase the frequency that this habitat is sampled.

Overall, the literature suggests that assessments of *Mysis* at night using pelagic gear, and their application to ecological processes (e.g. production, zooplanktivory), provide an incomplete picture of *Mysis* populations and their role in ecosystem structure and function.

### 3.2 Role of benthic food resources

Benthic food resources probably play a significant role in *Mysis* energy dynamics. *Mysis* consume and grow on a variety of benthic foods including detritus, zooplankton eggs, amphipods, and benthic zooplankton (Albertsson, 2004; Johannsson et al., 2001; Karlson & Viitasalo‐Frosen, 2009; Parker, 1980; Sierszen et al., 2011; Viitasalo & Viitasalo, 2004). For example, no differences in growth rates or survival were observed for *M. mixta* fed *Artemia* nauplii versus dried and ground plant material (*Enteromorpha* spp.), with growth rates similar to those observed in situ (Gorokhova & Hansson, 1999). *Neomysis americana* also grew well on a diet of cordgrass (*Spartina alterniflora*) detritus (Zagursky & Feller, 1985). Similar results were reported for mysids fed fresh and decaying plant material and detritus (Irvine, Moss, Bales, & Snook, 1993; Lasenby & Van Duyn, 1992; Lesutiene, Gorokhova, Gasiunaite, & Razinkovas, 2008; Speirs, Lawrie, Raffaelli, Gurney, & Emes, 2002). Ingestion rates of detritus were approximately double those of phytoplankton in laboratory feeding experiments, on a caloric basis, and suggested that detritus played a significant role in growth of Lake Tahoe *Mysis* (Morgan, 1979). Large *Mysis* captured in the pelagia of Lake Superior at night in September relied on benthic sources for 27–58% of their diet (Sierszen et al., 2011), *Mysis* from Okanagan Lake assimilated 4–59% of their carbon from benthic sources (Whall & Lasenby, 2009), and pelagic zooplankton could not solely support *Mysis* growth in Lake Ontario (Johannsson, Rudstam, & Lasenby, 1994), providing further evidence that benthic food resources contribute to assimilated energy. Detritus has been found in the stomachs of almost all species of mysids from freshwater to marine habitats (Mauchline, 1980; Takahashi, 2004). Such results are not surprising, as mysids are efficient at digesting detritus and contain gut enzymes needed to breakdown plant material (Foulds & Mann, 1978; Friesen, Mann, & Novitsky, 1986; Wainwright & Mann, 1982). Consequently, more consideration and better estimates of daytime foraging are needed to fully realise the contribution of benthic resources to mysid growth and survival, and by extension, the influence of mysids on ecosystems.

Production estimates need to take into account the demographic distribution of mysids in both pelagic and benthic habitats, and the resources in those habitats including detritus and possibly benthic macro‐ and meio‐fauna (Karlson & Viitasalo‐Frosen, 2009). Growth and mortality estimates based solely on pelagic samples may under‐ and overestimate rates, respectively, because larger (and gravid) individuals of a population may disproportionately occupy benthic habitat at night (Figure 3). Furthermore, the lower temperature in bottom waters may increase energy conversion efficiency and lipid content and hence energy density of mysids (Chess & Stanford, 1999).

### 3.3 Benthic behaviour

In addition to mysids’ ability to consume benthic resources, *Mysis* probably use benthic habitat to further reduce their vulnerability to visual predators. For example, bloater (*Coregonus hoyi*) capture success rate for *Mysis* off bottom was nearly double that when *Mysis* were on bottom (Crowder & Binkowski, 1983). The sediment surface is used by *Mysis* to propel away from predators with greater acceleration and maximum speed than possible in pelagic habitat and to maintain their position on top of sediment in strong currents, and they can also burrow into the sediment (Bowers, 1988; Bowers, Cooper, & Hall, 1990; O'Malley, Dillon, et al., 2018; Robertson, Powers, & Anderson, 1968; Sellers, 1995). Deepwater sculpin (*Myoxocephalus thompsonii*) and other sculpin species (*Cottus bairdi*,* Cottus ricei*,* Cottus cognatus*), the primary demersal fishes that prey upon mysids in the Laurentian Great Lakes (Gamble, Hrabik, Stockwell, et al., 2011; Gamble, Hrabik, Yule, et al., 2011; Hondorp, Pothoven, & Brandt, 2011; Weidel et al., 2017), consume prey on or in the sediment (Kraft & Kitchell, 1986; Selgeby, 1988) using vibration to detect prey (Hoekstra & Janssen, 1985; Janssen, 1990). Such a foraging strategy, however, may be relatively ineffective on *Mysis* (Bowers, 1988) given sculpins’ capture success rates of only *c*. 10% for *Mysis* compared to 45 and 78% for less agile amphipod and chironomid prey, respectively (Hondorp, 2006). In systems where dreissenid mussels have colonised and expanded into *Mysis* benthic habitat, the additional complex shell habitat and organic matter could influence *Mysis* interaction with predators and sediments (Stewart, Miner, & Lowe, 1998), but this remains to be tested.

*Mysis* behaviour on and in sediment may also influence benthic habitat biogeochemistry. For example, perturbation of sediments by *Mysis* burrowing into or disturbing the sediment surface can increase oxygen transport across the diffusive boundary layer and reduce the growth of sulfur‐producing bacteria typical of anoxic conditions (Lindström & Sandberg‐Kilpi, 2008), and thus potentially contribute to and impact benthic–pelagic fluxes in organic matter, metabolites, and nutrients (Karlson, Hulth, Ringdahl, & Rosenberg, 2005; Kristensen, 2000; Lohrer, Thrush, & Gibbs, 2004). In systems where benthic invertebrates may be reduced (e.g. *Diporeia* in the Laurentian Great Lakes; Nalepa, Fanslow, Pothoven, Foley, & Lang, 2007), bio‐perturbation by *Mysis* could play an increasingly important role in biogeochemical processes at the sediment–water interface. Similarly, in systems where nonnative dreissenid mussels have increased sedimentation rates and sediment organic matter content (e.g. Klerks, Fraleigh, & Lawniczak, 1996; Stewart et al., 1998), the importance of *Mysis*‐sediment dynamics will probably be greater than in systems without deep, filter‐feeding mussels.

### 3.4 Food web and ecosystem effects

*Mysis* use of benthic food resources probably has compounding effects. The total energy consumption by *Mysis* populations may be underestimated if based on pelagic feeding rates. When energy consumption is inferred from growth rates and bioenergetics models, part of that energy intake will be from benthic resources and, if not accounted for, will bias high the inferred effects on zooplankton. Thus, the pathways by which energy flows through mysid populations are probably different from the estimates and inferences drawn from pelagic‐focused literature (Johannsson et al., 1994; Lehtiniemi et al., 2002, 2009; Viherluoto, Kuosa, Flinkman, & Viitasalo, 2000). For example, estimates of *Mysis* consumption have generally assumed a diet of 100% zooplankton (Bunnell, Davis, Warner, Chriscinske, & Roseman, 2011; Chipps & Bennett, 2000; Gal et al., 2006; Hrycik et al., 2015; Murtaugh, 1984; Rudstam et al., 1989; Rudstam, Hansson, Johansson, & Larsson, 1992; but see Stewart & Sprules, 2011). The impact that *Mysis* can have on zooplankton community composition and size structure and the subsequent negative consequences for pelagic planktivorous fishes is unequivocal, as clearly seen in systems where *Mysis* have been introduced (Devlin et al., 2016; Lasenby et al., 1986; Nesler & Bergersen, 1991). However, estimates of *Mysis* zooplanktivory based on pelagic sampling, bioenergetics, and assumed consumption of 100% zooplankton will be biased high because these estimates under‐represent how much benthic energy flows through *Mysis*, and consequently the amount of benthic energy that flows through other components of the food web that directly or indirectly interact with *Mysis* (Johannsson et al., 1994, 2001; Viherluoto et al., 2000).

Benthic *Mysis* and benthic resources used by *Mysis* may explain why mass‐balance food web models often estimate greater fish predation on *Mysis* than observed *Mysis* production or biomass can support. *Mysis* biomass and production had to be increased 2–3‐fold over observed values to meet the estimated consumption demands of planktivorous fishes in a Lake Ontario model (Stewart & Sprules, 2011). Lake Michigan mass‐balance food web models also estimated fish predation of *Mysis* to be greater than *Mysis* biomass observations (Rogers, Bunnell, Madenjian, & Warner, 2014). Similarly, in the Baltic Sea, zooplankton production was insufficient to support the quantity of *Mysis* consumed by fishes (Harvey, Cox, Essington, Hansson, & Kitchell, 2003) when pelagic invertebrates were assumed to consume 100% zooplankton (Sandberg, Elmgren, & Wulff, 2000). To account for the imbalance, and based on results from stable isotope analyses (Hansson et al., 1997), a 50/50 diet balance of zooplankton and plant material for *Mysis* was needed in the food web model (Harvey et al., 2003). The evidence from the literature suggests such holes in mass balance food web models could be filled by accounting for *Mysis* in benthic habitats and their use of benthic resources.

**FIGURE 4:**
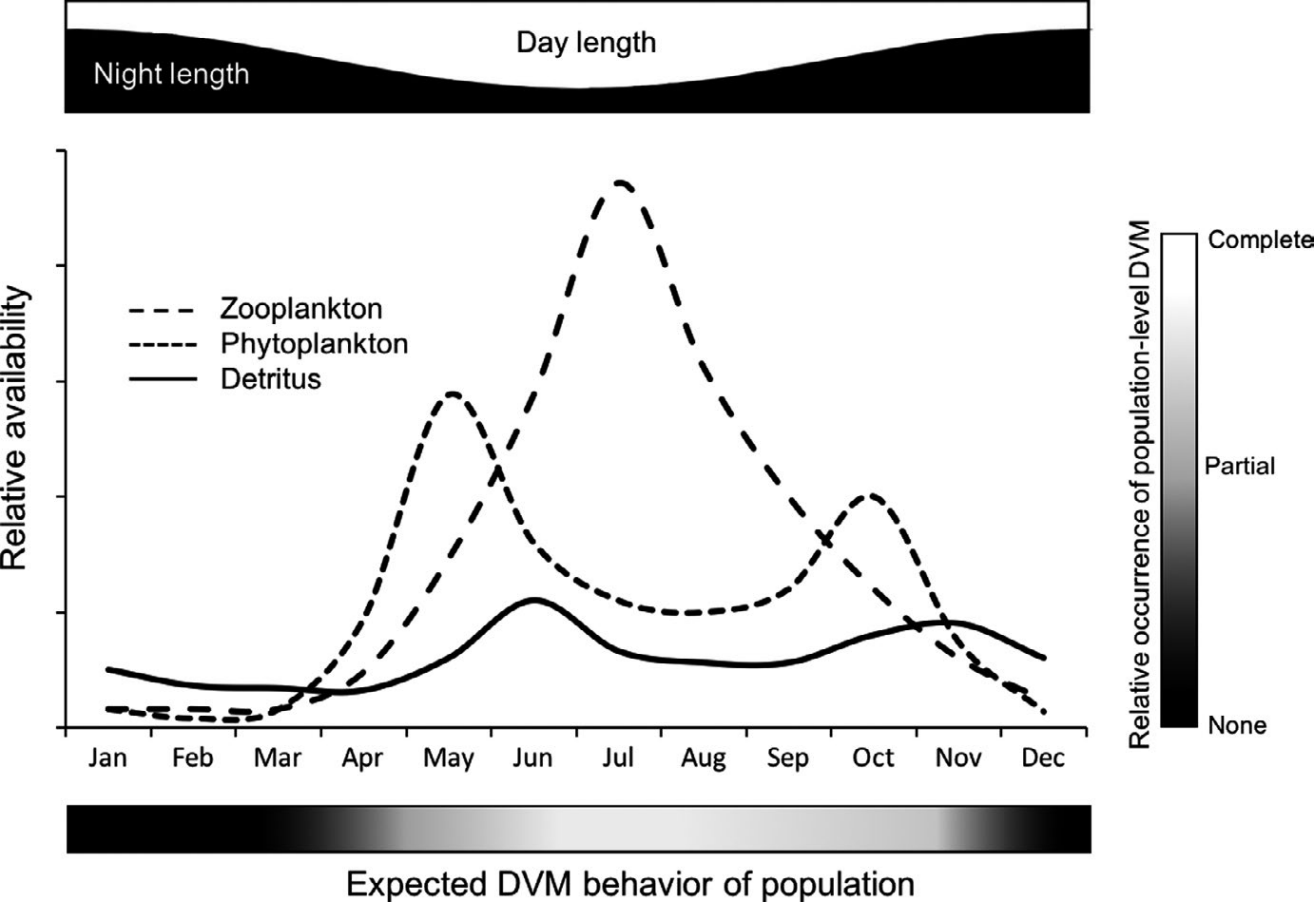
Expectations of diel vertical migration (DVM) behaviour of *Mysis* populations given seasonal availability of pelagic (following the Plankton Ecology Group model; Sommer et al., 2012) and benthic resources, and observations from Lake Ontario (Johannsson et al., 2001). Time constraints on *Mysis* foraging in pelagic habitats is indicated by day and night length in the upper panel

One consequence of the pelagic focus of *Mysis* research in freshwater systems is the implicit inference that pelagic production is the dominant energy pathway for *Mysis*. Our literature survey, however, suggests that *Mysis* may spend more time on the bottom than assumed. In winter, when pelagic production is low and *Mysis* fecundity is high, the motivation to migrate into the water column at night is also presumably low and a greater proportion of the population probably spends more time on bottom than during the other seasons (e.g. Johannsson et al., 2001; Salemaa et al., 1986; but see Lehtiniemi et al., 2009; Figure 4). Conversely, in summer when pelagic production is high, the motivation to migrate is also presumably high but short nights limit access to pelagic resources. Additionally, the abundance and quality of benthic resources is influenced by season and water column depth (Auer, Cannon, & Auer (2009); Eadie, Chambers, Gardner, & Bell, 1984; Ostrom, Long, Bell, & Beals, 1998; Scharold, Lozano, & Corry, 2004), suggesting dynamic cost:benefit trade‐offs to migration over space and time, which probably influences the amount of benthic feeding by mysids (Johannsson et al., 2001; Sierszen et al., 2011, 2014). However, when integrated over a year, the proportion of time and the proportion of mysids that occupy benthic habitat is probably greater than pelagic habitat. Because *Mysis* continuously feed when on bottom, regardless of time of day (O'Malley & Stockwell, 2019), detritus and other benthic resources are likely to compose a large portion of *Mysis* diets and thus energy processing and assimilation.

An underlying theme in *Mysis* research, either implicitly implied or explicitly stated by the focus on the pelagia, is that benthic resources act as a resource subsidy to support *Mysis* populations through periods of low pelagic production (Chipps & Bennett, 2000; Patwa, Christensen, Lasenby, Webster, & MacKay, 2007). An alternative theme is that pelagic production serves as a seasonal resource subsidy or provides a limiting nutrient for a foundational benthic energy pathway. The potential for benthic resources to be the dominant energy source for mysids, and the role *Mysis* may play in moving, distributing, and cycling benthic‐derived energy and nutrients at the sediment–water boundary and between benthic and pelagic habitats in lakes could be far greater than we thought, and remains a hypothesis to be tested.

## 4 RESEARCH QUESTIONS AND HYPOTHESES

Below, we identify a series of important questions and hypotheses that we believe will advance our understanding of the role of benthic habitat to *Mysis* ecology. We organised the questions under three themes: (1) what drives decisions to migrate or not; (2) the importance of benthic habitat for *Mysis* assessment; and (3) how benthic resources may impact *Mysis* dynamics in a broader ecosystem context.

### 4.1 (1) What body‐states and environmental conditions determine whether mysids migrate?

Conditions in benthic and pelagic habitats vary seasonally (Figure 4), as do mysids (e.g. life‐stages, body‐state). To understand mysid migration, we need to consider external and internal conditions (Nathan et al., 2008) and evaluate whether variation in migration behaviour at the population level is a result of evolutionarily developed life histories/behaviours or individual decision making. In this context, we pose a series of sub‐questions on processes and mechanisms that may drive emergent population‐level migration behaviours.

*(1a) Is partial DVM a result of fixed or plastic behaviours at the individual or group level?* Whether a *Mysis* population can be divided into sub‐groups that each behave consistently within groups but differently among groups, or *Mysis* behaviour is an outcome of individual choice in response to external conditions or internal states, remains unclear. Several early studies proposed such alternative behaviours (Morgan, 1980; Rybock, 1978), and more recent studies using stable isotopes and genetics have found suggestive but mixed results (Euclide et al., 2017; Ogonowski et al., 2013). Another possibility is the presence of personality traits that influence, or at least reflect, differences in movement, including migration (Chapman et al., 2011; Mettke‐Hofmann, Ebert, Schmidt, Steiger, & Stieb, 2005; Nilsson, Nilsson, Alerstam, & Backman, 2010; Sih & Watters, 2005). In situ tests of fixed or plastic DVM behaviour remain difficult in the absence of the technology to track individual *Mysis* behaviour through time, although laboratory experiments and agent‐based models are viable options (e.g. DeAngelis & Diaz, 2019; Langer et al., 2019).

*(1b) What is the relative importance of pelagic and benthic prey resources for Mysis growth? How is growth influenced by prey availability and nutritional quality between benthic and pelagic habitats and across seasons? Do individuals need to migrate to survive, grow, and reproduce?* Our synthesis suggests that a more diverse perspective is needed to better quantify the absolute and relative roles of benthic and pelagic prey resources for *Mysis* growth. However, observations that individuals captured in pelagic habitat during the day tend to be smaller, that smaller individuals move higher up in the water column at night, and larger individuals tend to dominate night benthic habitat (e.g. Beeton, 1960; Boscarino, Rudstam, Tirabassi, et al., 2010; O'Malley, Dillon, et al., 2018; Ogonowski et al., 2013; Salemaa et al., 1986) suggest that ontogeny and body‐state need to be incorporated into such studies.

*(1c) Are predation risks higher in benthic or pelagic habitats? Are DVM patterns dependent on Mysis density and the presumably closer proximity to predators when on the bottom (2‐D habitat) than in the water column (3‐D habitat)?* To our knowledge, studies on *Mysis* predation risk to fish have been primarily focused on pelagic settings (e.g. Boscarino, Rudstam, Tirabassi, et al., 2010; Jensen, Hrabik, Martell, Walters, & Kitchell, 2006; Levy, 1991; Mason & Patrick, 1993). More information is needed to explore risk/benefit aspects of staying on bottom (c.f. Crowder & Binkowski, 1983; Harrington, Hrabik, & Mensinger, 2015; Janssen, 1990) compared to the pelagia. Cannibalism (Fraser, Cahill, Lasenby, Mackay, & Milford, 2005; Quirt & Lasenby, 2002) also needs to be considered as part of the equation to migrate or not, especially as *Mysis* density can rapidly increase and concentrate in benthic habitats (O'Malley, Dillon, et al., 2018) compared to dispersal in a 3‐D pelagic environment. Modelling encounter rates between *Mysis* and their predators in 3‐D (Gerritsen & Strickler, 1977) and 2‐D (Hutchinson & Waser, 2007) foraging arenas may prove useful to this end.

### 4.2 (2) How much does the presence of benthic Mysis affect estimates of abundance and production?

Fundamental questions remain about the extent to which pelagic‐only sampling biases our inferences about *Mysis* population dynamics.

*(2a) What proportion of Mysis populations remain benthic at night? What proportion remain pelagic during the day?* The proportion of *Mysis* that remains on the bottom at night probably varies among sites, lakes, and seasons, as does the proportion that remains suspended in the pelagic zone during the day (O'Malley, Hansson, et al., 2018). Exploration of *Mysis* behaviour in extreme environments may provide useful insights as to what conditions may influence DVM behaviour. For example, our conceptual model predicts limited DVM behaviour in winter when pelagic resources are at annual lows (Figure 4). By extension, do *Mysis* populations exhibit restricted DVM behaviour in clear, high‐latitude oligotrophic systems, where benthic production in the littoral zone is the dominant source of system primary production (Ask et al., 2009; Sierszen, McDonald, & Jensen, 2003; Vadeboncoeur et al., 2003)? Conversely, do *Mysis* populations exhibit increased suspension in pelagic habitat during the day in dark‐water, productive systems (Ball, Mihuc, Myers, & Stockwell, 2015; Griffiths, 2007; Penk, 2011)?

*(2b) What are the demographic differences between benthic‐ and pelagic‐caught mysids, and how do such differences influence population production estimates?* Assessing demographic differences between benthic and pelagic habitats across bathymetric depths, time of day, and seasons will provide more accurate population assessments, as well as contribute to questions and hypotheses about the mechanisms driving partial DVM (see questions 1a–c above) and to fill in missing biomass and production in food web models (see *Food web and ecosystem effects* above).

### 4.3 (3) How does mysids’ use of benthic resources affect their ecological resistance to system change and their ecosystem function?

Zooplanktivory by *Mysis* can be intense and alter zooplankton community structure and function (Lasenby et al., 1986; Nesler & Bergersen, 1991). However, decreases in pelagic zooplankton may not necessarily induce a negative feedback in *Mysis* abundance or growth because they can exploit benthic resources (Chipps & Bennett, 2000) and thus exhibit a high degree of adaptive capacity with changing conditions (McMeans et al., 2016). For example, in addition to withstanding intense competition for pelagic zooplankton (Bunnell et al., 2011), *Mysis* may also be able to resist declines in system productivity as a result of oligotrophication (Barbiero, Lesht, & Warren, 2012) and shifts in energy flow from green (pelagic) to brown (benthic) pathways induced by invasive species (Vanderploeg, Liebig, Nalepa, Fahnenstiel, & Pothoven, 2010). The ability of mysids to use both pelagic and benthic resources probably serves as a buffer against declines in either resource, such that they can maintain their role as an energy conduit across habitats and trophic levels during periods of system change (Johannsson et al., 2001).

## 5 CONCLUSION

The disproportionate focus on the pelagic phase of *Mysis* DVM in the published literature and the likely, but perhaps under‐appreciated, role that benthic habitat plays in *Mysis* ecology suggests a complementary lens through which we should approach *Mysis* research. Partial DVM in freshwater mysids appears to be the norm rather than the exception. Thus, instead of the seemingly implicit assumption that the pelagic habitat is the most important habitat for *Mysis* and the benthic environment is simply a hiding place during daylight hours, we propose an alternative and perhaps provocative perspective that benthic habitat is equally, if not more, important than pelagic habitat to understanding *Mysis* ecology. Such a shift in perspective requires testing that focuses research more equally on the two habitats. A more‐balanced perspective will result in a better understanding of the drivers of *Mysis* DVM behaviour and yield new insights into the ecosystem effects of animals, such as mysids, that rely on both benthic and pelagic habitats.

## Supporting information

Tables S1–S3

## Data Availability

As this is a review paper there are no data.
