# Supplementary material for: Benthic habitat is an integral part of freshwater Mysis ecology
Source: Freshw Biol. 2020 Jul 23;65(11):1997–2009. doi: 10.1111/fwb.13594 (PMC7689720; doi:10.1111/fwb.13594)
Supplement: Supplementary file 1 — Tables S1–S3 [file FWB-65-1997-s001.docx]

Table S1. Literature survey results for identification of density, biomass, size-frequency distribution, mean size, growth rate, and life-stage of *Mysis* from field studies. The literature was initially searched using Web of Science, with terms “*Mysis*” AND “abundance OR densit* OR biomass* OR growth OR size OR length OR distribution” under the field “Topic” from 1945 to 2019. The search was performed on 26 July 2019. The search resulted in a total of 517 articles. Titles and abstracts were examined for relevance to demographics of freshwater *Mysis* spp. and *M. mixta* from field studies. Additionally, relevant papers were also examined for more references as papers from older journals or from journals and theses that were not available in Web of Science. Density also includes numbers or abundance. We did not include papers that developed new sampling devices (Lasenby & Sherman, 1991) or relied solely on acoustics without net samples (Levy, 1991; Miller, 2003). Papers that relied on previously published values (Morgan, 1985; Morgan, Threlkeld, & Goldman, 1978; Sell, 1982; Sullivan & Rudstam, 2016) were not included. Theses and dissertations were included unless data were published in peer-reviewed literature. In those cases (e.g., Morgan, 1985), information from the published papers was used. Papers which report a mix of original and previously reported values were included, but habitat was only recorded for original data (e.g., Goldman, Morgan, Threlkeld, & Angeli, 1979). Robertson, Powers, & Anderson (1968) was not included because density estimates were relative, based on visual observations. Studies that reported metrics but not methods were excluded (Adare & Lasenby, 1994; Lasenby & Langford, 1972). Habitat sampled: P = pelagic, B = benthic, PB = both pelagic and benthic. The full references of papers cited in Table S1 can be found in the supplementary references online only.

| Reference | Species | Density | Biomass | Size dist'n | Mean size | Growth rate | Life-stage |
| --- | --- | --- | --- | --- | --- | --- | --- |
| Adare (1992) | *M. relicta* | - | - | B | - | B | B |
| Ahrenstorff, Hrabik, Stockwell, Yule, & Sass (2011) | *M. diluviana* | P | - | - | - | - | - |
| Andrew & Woodward (1993) | *M. relicta* | PB | PB | - | - | PB | PB |
| Bagge, Liimatainen, & Liljaniemi (1996) | *M. relicta* | PB | - | - | - | - | - |
| Ball, Mihuc, Myers, & Stockwell (2015) | *M. diluviana* | P | P | P | P | P | P |
| Barker (1973) | *M. relicta* | - | - | PB | - | - | PB |
| Barz & Hirche (2009) | *M. mixta* | P | - | P | - | - | - |
| Beattie & Clancey (1991) | *M. relicta* | P | - | - | - | - | - |
| Beeton (1960) | *M. relicta* | P | - | - | - | - | P |
| Bergersen & Maiolie (1981) | *M. relicta* | B | - | - | - | - | - |
| Bergersen, Gregg, Maiolie, & Hamilton (1993) | *M. relicta* | PB | - | PB | - | PB | PB |
| Boscarino, Rudstam, Eillenberger, & O'Gorman (2009) | *M. diluviana* | P | P | - | P | P | - |
| Bowers (1988) | *M. relicta* | PB | - | - | - | - | P |
| Brown (1998) | *M. relicta* | P | - | P | - | - | - |
| Brownell (1970) | *M. relicta* | - | - | PB | - | - | PB |
| Bunnell, Davis, Warner, Chriscinske, & Roseman (2011) | *M. relicta* | P | P | - | - | - | - |
| Caldwell & Wilhelm (2012) | *M. diluviana* | P | P | P | P | P | P |
| Caldwell, Wilhelm, & Dux (2016) | *M. diluviana* | P | - | - | - | - | - |
| Carpenter, Mansey, & Watson (1974) | *M. relicta* | P | - | P | - | - | P |
| Chess & Stanford (1998) | *M. relicta* | - | - | - | P | P | P |
| Chipps & Bennett (1996) | *M. relicta* | P | - | P | - | - | P |
| Chipps & Bennett (2000) | *M. relicta* | P | - | P | P | P | P |
| Devlin et al. (2017) | *M. diluviana* | P | - | P | - | - | P |
| Eaton & Kardos (1973) | *M. relicta* | P | - | - | P | - | P |
| Ellis et al. (2011) | *M. diluviana* | P | - | - | - | - | - |
| Euclide, Hansson, & Stockwell (2017) | *M. diluviana* | - | - | - | PB | - | PB |
| France (2012) | *M. relicta* | P | - | - | - | - | - |
| Fürst (1972) | *M. relicta* | - | - | B | - | B | B |
| Gal, Rudstam, & Greene (1999a) | *M. relicta* | P | P | P | - | - | - |
| Gal, Loew, Rudstam, & Mohammadian (1999b) | *M. relicta* | P | - | - | - | - | - |
| Gal et al. (2006) | *M. relicta* | P | P | - | P | P | P |
| Goldman et al. (1979) | *M. relicta* | P | - | - | - | - | - |
| Gregg (1976) | *M. relicta* | PB | - | PB | PB | PB | PB |
| Griffiths (2007) | *M. relicta* | P | - | - | - | P | P |
| Griffiths et al. (2015) | *M. salemaai* | P | - | - | - | - | - |
| Grossnickle & Morgan (1979) | *M. relicta* | PB | - | - | - | - | - |
| Gutowski (1978) | *M. relicta* | P | P | P | P | P | P |
| Hakala (1978) | *M. relicta* | P | P | P | P | P | P |
| Hamilton (1971) | *M. relicta* | B | - | - | - | - | - |
| Sture Hansson, Larsson, & Johansson (1990) | *M. mixta* | - | P | - | - | - | - |
| Hesthagen (1973) | *M. mixta* | B | - | - | - | - | B |
| Holda et al. (2019) | *M. diluviana* | P | P | P | P | P | P |
| Holmquist (1963) | *M. relicta* | B | - | - | - | - | - |
| Horppila et al. (2003) | *M. relicta* | PB | - | - | PB | PB | PB |
| Houghton, Bronte, Paddock, & Janssen (2010) | *M. diluviana* | B | - | - | - | - | - |
| Hrycik et al. (2015) | *M. diluviana* | P | - | P | P | P | P |
| Hyatt, McQueen, & Ogden (2018) | *M. diluviana* | - | P | - | - | - | P |
| Isaac, Hrabik, Stockwell, & Gamble (2012) | *M. diluviana* | P | - | - | - | - | - |
| Jensen, Yurista, Hrabik, & Stockwell (2009) | *M. relicta* | P | - | P | - | - | - |
| Johannsson (1992) | *M. relicta* | P | P | P | P | P | P |
| Johannsson (1995) | *M. relicta* | P | P | P | P | P | P |
| Johannsson, Bowen, Holeck, & Walsh (2011) | *M. diluviana* | P | P | P | P | P | P |
| Johnson & Martinez (2012) | *M. diluviana* | P | P | - | - | - | - |
| Johnson, Pate, Silver, & Sharp (2018) | *M. diluviana* | P | - | P | - | - | P |
| Jokela, Arnott, & Beisner (2011) | *M. diluviana* | P | - | - | - | - | - |
| Juday & Birge (1927) | *M. relicta* | - | - | - | - | - | PB |
| Jude et al. (2018) | *M. diluviana* | P | P | P | - | - | P |
| Kane (1904) | *M. relicta* | B | - | - | - | - | B |
| Kelly et al. (2011) | *M. diluviana* | P | - | - | - | - | - |
| Kjellberg, Hessen, & Nilssen (1991) | *M. relicta* | PB | PB | - | PB | PB | PB |
| Köhn & Waterstraat (1990) | *M. relicta* | - | - | B | B | - | B |
| Koksvik, Reinertsen, & Koksvik (2009) | *M. relicta* | P | - | - | - | - | - |
| Kopache & Siegried (1982) | *M. relicta* | P | P | P | - | - | P |
| Langeland (1981) | *M. relicta* | P | - | - | - | - | - |
| Langeland (1988) | *M. relicta* | PB | P | PB | - | - | PB |
| Langeland, Carl, Hicks, & Monroe (1991a) | *M. relicta* | P | P | - | - | - | - |
| Langeland, Koksvik, & Nydal (1991b) | *M. relicta* | PB | - | - | - | - | - |
| Larkin (1948) | *M. relicta* | - | - | PB | - | PB | PB |
| Lasenby (1971) | *M. relicta* | PB | - | - | - | - | - |
| Lehman, Bowers, Gensemer, Warren, & Branstrator (1990) | *M. relicta* | P | - | - | - | - | - |
| Lehtiniemi, Viitasalo, & Kuosa (2002) | *M. mixta* | - | - | P | - | - | - |
| Lehtiniemi, Kiljunen, & Jones (2009) | *M. relicta, M. salemaai, M. mixta* | - | - | - | B | - | B |
| Liljendahl-Nurminen, Horppila, Uusitalo, & Niemistö (2008) | *M. relicta* | - | P | - | - | - | - |
| Madeira, Brooks, & Seale (1982) | *M. relicta* | - | P | - | - | - | - |
| Margoński & Maciejewska (1999) | *M. mixta* | P | P | - | - | - | - |
| Maxwell (2003) | *M. diluviana* | P | - | - | - | - | - |
| McCoy (2015) | *M. diluviana* | P | - | P | - | - | - |
| McDonald, Crowder, & Brandt (1990) | *M. relicta* | P | - | P | P | - | - |
| McWilliam (1970) | *M. relicta* | PB | - | PB | PB | - | PB |
| Moen & Langeland (1989) | *M. relicta* | PB | PB | PB | - | - | PB |
| Morgan (1976) | *M. relicta* | PB | PB | PB | - | PB | PB |
| Morgan (1980) | *M. relicta* | P | - | P | P | - | P |
| Morgan (1981) | *M. relicta* | P | - | P | P | P | P |
| Morgan & Beeton (1978) | *M. relicta* | B | - | B | - | B | B |
| Morgan & Threlkeld (1982 | *M. relicta* | P | - | P | P | - | P |
| Mundie (1959) | *M. relicta* | P | - | - | - | - | - |
| Naesje, Jensen, Moen, & Saksgård (1991) | *M. relicta* | PB | - | - | - | - | - |
| Naesje, Saksgard, Jensen, & Sandlund (2003) | *M. relicta* | PB | PB | PB | PB | - | PB |
| Nero & Davies (1982) | *M. relicta* | PB | - | - | - | - | PB |
| Nero & Schindler (1983) | *M. relicta* | P | - | - | P | P | P |
| Nero & Sprules (1986) | *M. relicta* | P | - | - | - | - | P |
| Nowicki et al. (2017) | *M. diluviana* | - | P | - | - | - | - |
| O'Malley, Dillon, Paddock, Hansson, & Stockwell (2018a) | *M. diluviana* | B | - | - | - | - | - |
| O'Malley, Hansson, & Stockwell (2018b) | *M. diluviana* | PB | - | PB | PB | - | PB |
| Ogonowski, Duberg, Hansson, & Gorokhova (2013a) | *M. salemaai* | - | - | - | - | PB | - |
| Ogonowski, Hansson, & Duberg (2013b) | *M. mixta, M. relicta* | P | P | P | - | - | - |
| Oliver et al. (2015) | *M. diluviana* | - | P | - | - | - | - |
| Paterson, Podemski, Wesson, & Dupuis (2011) | *M. diluviana* | P | - | P | P | P | P |
| Penk (2011) | *M. salemaai* | PB | - | - | PB | - | PB |
| Penk & Minchin (2014) | *M. salemaai* | P | - | - | - | - | - |
| Penk, Donohue, Minchin, & Irvine (2016) | *M. salemaai* | - | - | PB | PB | PB | PB |
| Penk, Donohue, & Irvine (2018) | *M. salemaai* | P | - | - | - | - | - |
| Pothoven & Vanderploeg (2004) | *M. relicta* | - | P | - | - | - | - |
| Pothoven, Fahnenstiel, Vanderploeg, & Luttenton (2000) | *M. relicta* | P | P | P | P | P | P |
| Pothoven, Fahnenstiel, & Vanderploeg (2004) | *M. relicta* | P | P | - | P | - | P |
| Pothoven, Fanslow, & Fahnenstiel (2012) | *M. diluviana* | P | - | P | P | P | P |
| Pothoven, Fahnenstiel, & Vanderploeg (2010) | *M. relicta* | P | - | P | P | P | P |
| Pothoven & Vanderploeg (2017) | *M. diluviana* | P | P | P | P | P | P |
| Ragnarsson-Stabo, Vrede, Axenrot, & Sandstrom (2014) | *Mysids* | P | - | - | - | - | - |
| Rennie et al. (2019) | *M. diluviana* | P | - | - | - | - | - |
| Reynolds & DeGraeve (1972) | *M. relicta* | B | B | B | B | B | B |
| Richards, Goldman, Frantz, & Wickwire (1975) | *M. relicta* | B | - | - | - | - | - |
| Rieman & Falter (1981) | *M. relicta* | P | - | - | - | - | - |
| Rosenberg (1972) | *M. mixta* | B | - | - | - | - | - |
| Rudstam, Hansson, & Larsson (1986) | *M. mixta, M. relicta* | P | - | - | P | P | P |
| Rudstam, Danielsson, Hansson, & Johansson (1989) | *M. mixta* | PB | - | - | PB | - | PB |
| Rudstam et al. (2008a) | *M. relicta* | P | - | P | P | - | - |
| Rudstam et al. (2008b) | *M. relicta* | P | P | P | - | - | - |
| Rybock (1978) | *M. relicta* | P | - | - | P | - | P |
| Salemaa, Tyystjarvimuuronen, & Aro (1986) | *M. relicta, M. mixta* | P | - | - | P | - | P |
| Salemaa, Vuorinen, & Välipakka (1990) | *M. mixta, M. relicta* | P | - | - | - | - | - |
| Särkkä (1972) | *M. relicta* | P | - | - | - | - | - |
| Scharf & Koschel (2004) | *M. relicta* | P | - | P | - | - | P |
| Scharf, Krappe, Koschel, & Waterstraat (2008) | *M. relicta* | P | - | - | - | - | - |
| Schindler et al. (2012) | *M. diluviana* | P | - | - | - | - | - |
| Schoen, Beauchamp, Buettner, & Overman (2015) | *M. diluviana* | P | - | - | - | - | - |
| Sellers (1995) | *M. relicta* | PB | - | - | - | - | - |
| Shea & Makarewicz (1989) | *M. relicta* | PB | P | P | P | P | P |
| Sierszen, Kelly, Corry, Scharold, & Yurista (2011) | *M. diluviana* | P | P | - | - | - | P |
| Silver et al. (2016) | *M. diluviana* | P | - | P | P | - | P |
| Simm & Kotta (1992) | *M. relicta* | P | - | - | - | - | - |
| Slife (2017) | *M. diluviana* | P | P | P | P | P | P |
| Smokorowski (1998) | *M. relicta* | P | - | P | P | P | P |
| Southern & Gardiner (1932) | *M. relicta* | P | - | - | - | - | - |
| Spencer, Potter, Bukantis, & Stanford (1999) | *M. relicta* | P | - | - | - | - | P |
| Vainola & Vainio (1998) | *M. relicta, M. mixta* | - | - | B | B | - | B |
| Van Duyn-Henderson & Lasenby (1986) | *M. relicta* | P | - | - | - | - | P |
| Viherluoto, Kuosa, Flinkman, & Viitasalo (2000) | *M. mixta, M. relicta* | - | - | - | P | P | - |
| Watkins et al. (2015) | *M. diluviana* | P | P | P | - | - | - |
| Wang, Consi, Hansen, & Janssen (2012) | *M. diluviana* | P | - | P | - | - | P |
| Waterstraat, Krappe, Riel, & Rumpf (2005) | *M. relicta* | PB | - | PB | - | - | PB |
| Zyblut (1967) | *M. relicta* | P | - | P | - | - | - |

Table S2. Literature survey results for visual identification of detritus in stomachs of *Mysis* from field studies. Literature was initially searched using Web of Science, with terms “*Mysis*” AND “diet OR gut OR stomach” under the field “Topic” from 1945 to 2019. The search was performed on 10 September 2019. The search resulted in a total of 244 articles. Titles and abstracts were examined for relevance to diet contents of freshwater *Mysis* spp. and *M. mixta* from field studies. Additionally, papers from the review by Grossnickle (1982) were included in survey results to expand the literature search before 1945. References in relevant papers were also examined for more references as papers from older journals or from journals and theses were not available in Web of Science. Habitat sampled: P = pelagic, B = benthic, PB = both pelagic and benthic. Detritus: Y = methods report detritus as a possible prey category and/or results report detritus as consumed; N = methods report detritus as a possible prey category and/or results report no detritus consumed; UNC = methods do not mention detritus as a possible prey category and results do not mention presence or absence of detritus (i.e., uncertain if detritus was present but not reported). Quantified: Q = detritus in gut was quantified, Pr = detritus in gut was qualitatively reported as present, N/A = not applicable because detritus was not consumed (Detritus = N) or detritus was not reported as present or absent (Detritus = UNC). The full references of papers cited in Table S2 can be found in the supplementary references online only.

| Reference | Species | Habitat  sampled | Detritus | Quantified |
| --- | --- | --- | --- | --- |
| Branstrator & Lehman (1991) | *M. relicta* | P | UNC | N/A |
| Caldwell et al. (2016) | *M. diluviana* | P | UNC | N/A |
| Chess & Stanford (1998) | *M. relicta* | P | Y | Pr |
| Folt, Rybock, & Goldman (1982) | *M. relicta* | P | Y | Pr |
| Forbes (1882) | *M. oculatus* | B | UNC | N/A |
| Green (1965) | *M. relicta* | B | UNC | N/A |
| Grossnickle (1982) | *M. relicta* | P | UNC | N/A |
| Sture Hansson et al. (1990) | *M. mixta* | P | UNC | N/A |
| Hrycik et al. (2015) | *M. diluviana* | P | UNC | N/A |
| Johannsson et al. (2001) | *M. diluviana* | PB | UNC | N/A |
| Kinsten & Olsén (1981) | *M. relicta* | PB | Y | Pr |
| Köhn & Waterstraat (1990) | *M. relicta* | B | Y | Q |
| Kopache & Siegried (1982) | *M. relicta* | P | UNC | N/A |
| Langeland (1981) | *M. relicta* | P | UNC | N/A |
| Langeland (1988) | *M. relicta* | PB | Y | Pr |
| Lasenby & Langford (1973) | *M. relicta* | PB | Y | Pr |
| Lasenby & Shi (2004) | *M. relicta* | PB | Y | Pr |
| Lehtiniemi et al. (2002) | *M. mixta* | PB | Y | Q |
| Lehtiniemi et al. (2009) | *M. relicta, M. salemaai, M. mixta* | B | Y | Q |
| McWilliam (1970) | *M. relicta* | PB | Y | Pr |
| Nordin, Arts, Johannsson, & Taylor (2008) | *M. relicta* | P | Y | Pr |
| O'Malley & Stockwell (2019) | *M. diluviana* | PB | Y | Q |
| O'Malley & Bunnell (2014) | *M. diluviana* | P | Y | Pr |
| O'Malley, Rudstam, Watkins, Holda, & Weidel (2017) | *M. diluviana* | P | UNC | N/A |
| Penk et al. (2018) | *M. salemaai* | P | UNC | N/A |
| Rudstam et al. (1989) | *M. mixta* | PB | Y | Q |
| Rybock (1978) | *M. relicta* | P | Y | Pr |
| Scharf & Koschel (2005) | *M. relicta* | P | Y | Q |
| Threlkeld, Rybock, Morgan, Folt, & Goldman (1980) | *M. relicta* | P | UNC | N/A |
| Viherluoto et al. (2000) | *M. relicta* | PB | Y | Q |
| Whall & Lasenby (2009) | *M. diluviana* | P | UNC | N/A |

Table S3. Literature survey results for experiments using *Mysis*. Literature was initially searched using Web of Science, with terms “Mysis” AND “feeding OR predation” AND “experiment” under the field “Topic” from 1945 to 2019. A search of Web of Science with terms “Mysis” AND “contaminant* OR toxi*” was also conducted. The searches were performed on 26 July 2019. The search resulted in a total of 164 articles. Titles and abstracts were examined for relevance to laboratory and field experiments using freshwater *Mysis* spp. and *M. mixta*. Habitat sampled: P = pelagic, B = benthic, PB = both pelagic and benthic, UNK = not reported. Prey categories: Y = used in experiment(s), N = not used in experiment(s), “-“ = prey category was reported at higher taxonomic level (zooplankton community). The full references of papers cited in Table S3 can be found in the supplementary references online only.

| Reference | Species | Habitat | Zoop comm | Other clad | Daphnia | Copepods | Rotifers | Zoop eggs | Amphipod | Mysis | Algae | Detritus | Brine shrimp | Artificial food |
| --- | --- | --- | --- | --- | --- | --- | --- | --- | --- | --- | --- | --- | --- | --- |
| Albertsson (2004) | *M. relicta, M. mixta* | B | Y | - | - | - | - | N | Y | N | N | Y | N | N |
| Bailey, Dick, Elwood, & MacNeil (2006) | *M. relicta* | B | N | N | N | N | N | N | Y | N | N | N | N | N |
| Barrios-O'Neill et al. (2014a) | *M. salemaai* | UNK | N | N | Y | N | N | N | N | N | N | N | N | N |
| Barrios-O'Neill, Dick, Ricciardi, MacIsaac, & Emmerson (2014b) | *M. salemaai* | P | N | N | Y | N | N | N | N | N | N | N | N | N |
| Berrill & Lasenby (1983) | *M. relicta* | UNK | N | N | N | N | N | N | N | N | N | N | Y | Y |
| Boscarino et al. (2007) | *M. relicta* | P | N | N | Y | N | N | N | N | N | N | N | N | N |
| Bowers & Grossnickle (1978) | *M. relicta* | P | N | N | N | N | N | N | N | N | Y | N | N | N |
| Bowers & Vanderploeg (1982) | *M. relicta* | P | Y | - | - | - | - | N | N | N | N | N | N | N |
| Chess & Stanford (1999) | *M. relicta* | P | Y | - | - | - | - | N | N | N | N | N | N | N |
| Chipps (1998) | *M. relicta* | P | N | N | Y | Y | N | N | N | N | N | N | N | N |
| Chipps & Bennett (2002) | *M. relicta* | UNK | N | N | Y | N | N | N | N | N | N | N | N | N |
| Cooper & Goldman (1980) | *M. relicta* | P | Y | - | - | - | - | N | N | N | N | N | N | N |
| Cooper & Goldman (1982) | *M. relicta* | P | N | N | N | Y | N | N | N | N | N | N | N | N |
| DeGraeve & Reynolds (1975) | *M. relicta* | PB | N | N | N | N | N | N | N | N | N | N | Y | Y |
| Dick et al. (2013) | *M. salemaai, M. diluviana* | UNK | N | Y | Y | Y | N | N | Y | N | N | N | N | N |
| Engström-Ost, Lehtiniemi, Green, Kozlowsky-Suzuki, & Viitasalo (2002) | *M. relicta* | PB | N | N | N | Y | N | N | N | N | N | N | N | N |
| Engström, Viherluoto, & Viitasalo (2001) | *M. mixta* | PB | N | N | N | Y | N | N | N | N | Y | N | N | N |
| Euclide & Stockwell (2015) | *M. diluviana* | PB | N | N | N | N | N | N | N | N | Y | N | Y | N |
| Folt et al. (1982) | *M. relicta* | P | N | N | N | Y | N | N | N | N | N | N | N | N |
| Gorokhova (1998) | *M. mixta* | P | N | N | N | N | N | N | N | N | N | N | Y | N |
| Gorokhova (2002) | *M. mixta* | UNK | Y | - | - | - | - | N | N | N | N | N | Y | N |
| Gorokhova (2009) | *M. mixta* | P | N | N | N | N | N | N | N | N | Y | N | Y | N |
| Gorokhova & Hansson (1997) | *M. mixta* | UNK | N | N | Y | N | N | N | N | N | N | N | Y | N |
| Gorokhova & Hansson (1999) | *M. mixta* | UNK | N | N | N | N | N | N | N | N | N | Y | Y | N |
| Gorokhova & Hansson (2000) | *M. mixta* | P | N | N | N | N | N | N | N | N | N | N | Y | N |
| Gorokhova & Lehtiniemi (2007) | *M. relicta, M. mixta* | P | N | Y | N | N | N | N | N | N | N | N | N | N |
| Grossnickle (1978) | *M. relicta* | P | N | Y | Y | Y | N | N | N | N | Y | N | N | N |
| Hamrén & Hansson (1999) | *M. mixta* | UNK | N | N | N | N | N | N | N | N | N | N | Y | N |
| (Hansson, De Stasio, Gorokhova, & Mohammadian (2001) | *M. mixta* | P | Y | - | Y | - | - | N | N | N | N | N | Y | N |
| (Iacarella, Dick, Alexander, & Ricciardi (2015) | *M. diluviana* | UNK | N | N | Y | N | N | N | N | N | N | N | N | N |
| Johannsson et al. (2008) | *M. diluviana* | UNK | N | N | N | N | N | N | N | N | N | N | Y | Y |
| Karlson & Viitasalo-Frosen (2009) | *M. relicta, M. mixta* | B | N | N | N | N | N | Y | N | N | N | Y | N | N |
| Kinsten & Olsén (1981) | *M. relicta* | UNK | Y | - | - | - | - | N | N | N | N | N | N | N |
| Klump, Kaster, & Sierszen (1991) | *M. relicta* | UNK | N | N | N | N | N | N | N | N | N | Y | N | N |
| Landrum, Frez, & Simmons (1992) | *M. relicta* | P | N | N | Y | N | N | N | N | N | Y | N | N | N |
| Lasenby & Fürst (1981) | *M. relicta* | UNK | Y | - | - | Y | - | N | N | N | N | N | N | N |
| Lehtiniemi & Lindén (2006) | *M. relicta, M. mixta* | P | Y | - | Y | Y | Y | N | N | N | N | N | N | N |
| Lester & McIntosh (1994) | *M. relicta* | P | N | N | N | Y | N | N | N | N | N | Y | N | N |
| Lindén & Kuosa (2004) | *M. relicta, M. mixta* | PB | N | N | N | N | N | N | N | N | Y | N | N | N |
| MacNeil & Dick (2012) | *M. relicta* | B | N | N | N | N | N | N | Y | N | N | N | N | N |
| Mohammadian, Hansson, & DeStasio (1997) | *M. mixta* | P | Y | - | - | - | - | N | N | N | N | N | N | N |
| Nero & Sprules (1986) | *M. relicta* | P | Y | - | - | - | - | N | N | N | N | N | N | N |
| Parker (1980) | *M. relicta* | B | N | N | N | N | N | N | Y | N | N | Y | N | N |
| Penk et al. (2018) | *M. salemaai* | UNK | N | N | Y | Y | N | N | N | N | N | N | N | N |
| Quirt & Lasenby (2002) | *M. relicta* | UNK | N | N | N | N | N | N | N | Y | N | N | N | N |
| Ramcharan & Sprules (1986) | *M. relicta* | UNK | N | N | Y | Y | N | N | N | N | N | N | N | N |
| Ramcharan, Sprules, & Nero (1985) | *M. relicta* | UNK | N | N | Y | Y | N | N | N | N | N | N | N | N |
| Rudstam, Hetherington, & Mohammadian (1999) | *M. relicta* | P | N | N | N | N | N | N | N | N | N | N | Y | N |
| Rybock (1978) | *M. relicta* | P | Y | - | - | - | - | N | N | N | N | N | N | N |
| Seckar (2009) | *M. relicta* | UNK | Y | - | - | - | - | N | N | N | N | N | N | N |
| Smokorowski (1998) | *M. relicta* | UNK | Y | - | - | - | - | N | N | N | N | N | N | N |
| Spencer et al. (1999) | *M. relicta* | UNK | Y | - | - | - | - | N | N | N | N | N | N | N |
| Van Duyn-Henderson & Lasenby (1986) | *M. relicta* | UNK | N | N | Y | Y | N | N | N | N | N | Y | N | N |
| Viherluoto & Viitasalo (2001a) | *M. mixta* | P | N | N | N | Y | N | N | N | N | N | N | N | N |
| Viherluoto & Viitasalo (2001b) | *M. mixta* | P | Y | - | - | - | - | N | N | N | N | N | N | N |
| Viitasalo (2007) | *M. mixta* | B | N | N | N | N | N | Y | N | N | N | N | N | N |
| Viitasalo & Viitasalo (2004) | *M. relicta, M. mixta* | B | N | Y | N | N | N | Y | N | N | N | N | N | N |
| Whall & Lasenby (2009) | *M. diluviana* | P | Y | - | - | - | - | N | N | N | N | N | N | N |

#

**Supplementary References**

Adare, K. I. (1992). *Seasonal changes in the total lipid content and life cycle characteristics of the freshwater shrimp, Mysis relicta.* (MSc), Trent University, Peterborough, ON, CA.

Adare, K. I., & Lasenby, D. C. (1994). Seasonal changes in the total lipid-content of the opossum shrimp, *Mysis relicta* (Malacostraca, Mysidacea). *Canadian Journal of Fisheries and Aquatic Sciences, 51*(9), 1935-1941. doi:DOI 10.1139/f94-195

Ahrenstorff, T. D., Hrabik, T. R., Stockwell, J. D., Yule, D. L., & Sass, G. G. (2011). Seasonally dynamic diel vertical migrations of *Mysis diluviana*, coregonine fishes, and siscowet lake trout in the pelagia of western Lake Superior. *Transactions of the American Fisheries Society, 140*(6), 1504-1520. doi:10.1080/00028487.2011.637004

Albertsson, J. (2004). Trophic interactions involving mysid shrimps (Mysidacea) in the near-bottom habitat in the Baltic Sea. *Aquatic Ecology, 38*(3), 457-469. doi:10.1023/B:Aeco.0000035163.30037.38

Andrew, T. E., & Woodward, E. (1993). Some observations on the populations of *Mysis relicta* in Lough Neagh. In R. B. Wood & R. V. Smith (Eds.), *Lough Neagh* (pp. 327-338). Netherlands: Kluwer Academic Publishers.

Bagge, P., Liimatainen, H. M., & Liljaniemi, P. (1996). Comparison of sampling methods for semipelagic animals in two deep basins of Lake Saimaa. *Hydrobiologia, 322*(1-3), 293-300. doi:10.1007/Bf00031842

Bailey, R. J. E., Dick, J. T. A., Elwood, R. W., & MacNeil, C. (2006). Predatory interactions between the invasive amphipod *Gammarus tigrinus* and the native opossum shrimp *Mysis relicta*. *Journal of the North American Benthological Society, 25*(2), 393-405. doi:10.1899/0887-3593(2006)25[393:Pibtia]2.0.Co;2

Ball, S. C., Mihuc, T. B., Myers, L. W., & Stockwell, J. D. (2015). Ten-fold decline in *Mysis diluviana* in Lake Champlain between 1975 and 2012. *Journal of Great Lakes Research, 41*(2), 502-509. doi:10.1016/j.jglr.2015.03.002

Barker, J. M. (1973). *Changes in the total energy content of Mysis relicta Lovén during its vertical migration*. Paper presented at the Proceedings of the 16th Conference on Great Lakes Research.

Barrios-O'Neill, D., Dick, J. T., Emmerson, M. C., Ricciardi, A., MacIsaac, H. J., Alexander, M. E., & Bovy, H. C. (2014a). Fortune favours the bold: a higher predator reduces the impact of a native but not an invasive intermediate predator. *Journal of Animal Ecology, 83*(3), 693-701. doi:10.1111/1365-2656.12155

Barrios-O'Neill, D., Dick, J. T. A., Ricciardi, A., MacIsaac, H. J., & Emmerson, M. C. (2014b). Deep impact: *in situ* functional responses reveal context-dependent interactions between vertically migrating invasive and native mesopredators and shared prey. *Freshwater Biology, 59*(10), 2194-2203. doi:10.1111/fwb.12423

Barz, K., & Hirche, H. J. (2009). Ecology of mysid shrimps in the Bornholm Basin (central Baltic Sea). *Helgoland Marine Research, 63*(4), 317-326. doi:10.1007/s10152-009-0160-0

Beattie, W. D., & Clancey, P. T. (1991). Effects of *Mysis relicta* on the zooplankton community and kokanee population of Flathead Lake, Montana. *American Fisheries Society Symposium, 9*, 39-48.

Beeton, A. M. (1960). The vertical migration of *Mysis relicta* in Lakes Huron and Michigan. *Journal of the Fisheries Research Board of Canada, 17*(4), 517-539.

Bergersen, E.P., Gregg, R.E., Maiolie, M.A., Hamilton, K., 1993. Mysids. Pages 123–134 in Aquatic ecology studies of Twin Lakes, Colorado, 1971– 1986. U.S. Bureau of Reclamation, Engineering and Science Monograph 43, Denver, CO, USA.

Bergersen, E. P., & Maiolie, M. (1981). *Mysis population estimates: A photographic technique*. Applied Sciences Branch, Division of Research, Engineering and Research Center, US Department of the Interior, Bureau of Reclamation.

Berrill, M., & Lasenby, D. C. (1983). Life-cycles of the freshwater mysid shrimp *Mysis relicta* reared at two temperatures. *Transactions of the American Fisheries Society, 112*(4), 551-553. doi:10.1577/1548-8659(1983)112<551:Lcotfm>2.0.Co;2

Boscarino, B. T., Rudstam, L. G., Eillenberger, J. L., & O'Gorman, R. (2009). Importance of light, temperature, zooplankton and fish in predicting the nighttime vertical distribution of *Mysis diluviana*. *Aquatic Biology, 5*(3), 263-279. doi:10.3354/ab00161

Bowers, J. A. (1988). Diel vertical migration of the opossum shrimp *Mysis relicta* in Lake Superior - observations and sampling from the Johnson-Sea-Link II submersible. *Bulletin of Marine Science, 43*(3), 730-738.

Bowers, J. A., & Grossnickle, N. E. (1978). The herbivorous habits of *Mysis relicta* in Lake Michigan. *Limnology and Oceanography, 23*(4), 767-776. doi:10.4319/lo.1978.23.4.0767

Bowers, J. A., & Vanderploeg, H. A. (1982). In situ predatory behavior of *Mysis relicta* in Lake Michigan. *Hydrobiologia, 93*(1-2), 121-131. doi:10.1007/Bf00008105

Branstrator, D. K., & Lehman, J. T. (1991). Invertebrate predation in Lake Michigan - regulation of *Bosmina longirostris* by *Leptodora kindtii*. *Limnology and Oceanography, 36*(3), 483-495. doi:10.4319/lo.1991.36.3.0483

Brown, R. H. (1998). *Abundance and distribution of Mysis relicta in Moosehead Lake, Maine*. Fishery progress report No. 98-2. Maine Department of Inland Fisheries and Wildlife, Augusta, ME, USA.

Brownell, W. N. (1970). *Studies on the ecology of Mysis relicta in Cayuga Lake.* (MSc), Cornell University, Ithaca, N. Y.

Bunnell, D. B., Davis, B. M., Warner, D. M., Chriscinske, M. A., & Roseman, E. F. (2011). Planktivory in the changing Lake Huron zooplankton community: *Bythotrephes* consumption exceeds that of *Mysis* and fish. *Freshwater Biology, 56*(7), 1281-1296. doi:10.1111/j.1365-2427.2010.02568.x

Caldwell, T. J., & Wilhelm, F. M. (2012). The life history characteristics, growth and density of *Mysis diluviana* in Lake Pend Oreille, Idaho, USA. *Journal of Great Lakes Research, 38*, 58-67. doi:10.1016/j.jglr.2011.07.010

Caldwell, T. J., Wilhelm, F. M., & Dux, A. (2016). Non-native pelagic macroinvertebrate alters population dynamics of herbivorous zooplankton in a large deep lake. *Canadian Journal of Fisheries and Aquatic Sciences, 73*(5), 832-843. doi:10.1139/cjfas-2015-0144

Carpenter, G. F., Mansey, E. L., & Watson, N. H. F. (1974). Abundance and life history of *Mysis relicta* in the St. Lawrence Great Lakes. *Journal of the Fisheries Research Board of Canada, 31*(3), 319-325. doi:10.1139/f74-051

Chess, D. W., & Stanford, J. A. (1998). Comparative energetics and life cycle of the opossum shrimp (*Mysis relicta*) in native and non-native environments. *Freshwater Biology, 40*(4), 783-794. doi:10.1046/j.1365-2427.1998.00373.x

Chess, D. W., & Stanford, J. A. (1999). Experimental effects of temperature and prey assemblage on growth and lipid accumulation by *Mysis relicta* Lovén. *Hydrobiologia, 412*, 155-164. doi:10.1023/A:1003886920400

Chipps, S. R. (1998). Temperature-dependent consumption and gut-residence time in the opossum shrimp *Mysis relicta*. *Journal of Plankton Research, 20*(12), 2401-2411. doi:10.1093/plankt/20.12.2401

Chipps, S. R., & Bennett, D. H. (1996). Comparison of net mesh sizes for estimating abundance of the opossum shrimp *Mysis relicta* from vertical hauls. *North American Journal of Fisheries Management, 16*(3), 689-692.

Chipps, S. R., & Bennett, D. H. (2000). Zooplanktivory and nutrient regeneration by invertebrate (*Mysis relicta*) and vertebrate (*Oncorhynchus nerka*) planktivores: Implications for trophic interactions in oligotrophic lakes. *Transactions of the American Fisheries Society, 129*(2), 569-583. doi:10.1577/1548-8659(2000)129<0569:Zanrbi>2.0.Co;2

Chipps, S. R., & Bennett, D. H. (2002). Evaluation of a *Mysis* bioenergetics model. *Journal of Plankton Research, 24*(1), 77-82. doi:10.1093/plankt/24.1.77

Cooper, S. D., & Goldman, C. R. (1980). Opossum shrimp (*Mysis relicta*) predation on zooplankton. *Canadian Journal of Fisheries and Aquatic Sciences, 37*(6), 909-919. doi:10.1139/f80-120

Cooper, S. D., & Goldman, C. R. (1982). Environmental factors affecting predation rates of *Mysis relicta*. *Canadian Journal of Fisheries and Aquatic Sciences, 39*(1), 203-208. doi:10.1139/f82-024

DeGraeve, G. M., & Reynolds, J. B. (1975). Feeding behavior and temperature and light tolerance of *Mysis relicta* in the laboratory. *Transactions of the American Fisheries Society, 104*(2), 394-397. doi:10.1577/1548-8659(1975)104<394:FBATAL>2.0.CO;2

Devlin, S. P., Tappenbeck, S. K., Craft, J. A., Tappenbeck, T. H., Chess, D. W., Whited, D. C., . . . Stanford, J. A. (2017). Spatial and temporal dynamics of invasive freshwater shrimp (*Mysis diluviana*): Long-term effects on ecosystem properties in a large oligotrophic lake. *Ecosystems, 20*(1), 183-197. doi:10.1007/s10021-016-0023-x

Dick, J. T. A., Gallagher, K., Avlijas, S., Clarke, H. C., Lewis, S. E., Leung, S., . . . Ricciardi, A. (2013). Ecological impacts of an invasive predator explained and predicted by comparative functional responses. *Biological Invasions, 15*(4), 837-846. doi:10.1007/s10530-012-0332-8

Eaton, S. W., & Kardos, L. P. (1973). *Mysis* and some other large invertebrates of Canandaigua Lake, 1972. *Science Studies, St. Bonaventure University, 29*, 63-75.

Ellis, B. K., Stanford, J. A., Goodman, D., Stafford, C. P., Gustafson, D. L., Beauchamp, D. A., . . . Hansen, B. S. (2011). Long-term effects of a trophic cascade in a large lake ecosystem. *Proceedings of the National Academy of Sciences of the United States of America, 108*(3), 1070-1075. doi:10.1073/pnas.1013006108

Engström, J., Viherluoto, M., & Viitasalo, M. (2001). Effects of toxic and non-toxic cyanobacteria on grazing, zooplanktivory and survival of the mysid shrimp *Mysis mixta*. *Journal of Experimental Marine Biology and Ecology, 257*(2), 269-280. doi:10.1016/s0022-0981(00)00339-7

Engström-Ost, J., Lehtiniemi, M., Green, S., Kozlowsky-Suzuki, B., & Viitasalo, M. (2002). Does cyanobacterial toxin accumulate in mysid shrimps and fish via copepods? *Journal of Experimental Marine Biology and Ecology, 276*(1-2), 95-107. doi:10.1016/S0022-0981(02)00241-1

Euclide, P. T., Hansson, S., & Stockwell, J. D. (2017). Partial diel vertical migration in an omnivorous macroinvertebrate, *Mysis diluviana*. *Hydrobiologia, 787*(1), 387-396. doi:10.1007/s10750-016-2982-5

Euclide, P. T., & Stockwell, J. D. (2015). Effects of gut content on δ15N, δ13C and C:N of the macroinvertebrate *Mysis diluviana*. *Journal of Great Lakes Research, 41*(3), 926-929. doi:10.1016/j.jglr.2015.05.002

Folt, C. L., Rybock, J. T., & Goldman, C. R. (1982). The effect of prey composition and abundance on the predation rate and selectivity of *Mysis relicta*. *Hydrobiologia, 93*(1-2), 133-143. doi:10.1007/Bf00008106

Forbes, S. A. (1882). On some entomostraca of Lake Michigan and adjacent waters. *American Naturalist, 16*(7), 640-649.

France, R. L. (2012). Omnivory, vertical food-web structure and system productivity: stable isotope analysis of freshwater planktonic food webs. *Freshwater Biology, 57*(4), 787-794. doi:10.1111/j.1365-2427.2012.02744.x

Fürst, M. (1972). Livscykler, tillväxt och reproduktion hos *Mysis relicta* Lovén. *Information fràn Sötvattenslaboratoriet, Drottningholm, 11*, 1-41.

Gal, G., Loew, E. R., Rudstam, L. G., & Mohammadian, A. M. (1999b). Light and diel vertical migration: spectral sensitivity and light avoidance by *Mysis relicta*. *Canadian Journal of Fisheries and Aquatic Sciences, 56*(2), 311-322. doi:10.1139/cjfas-56-2-311

Gal, G., Rudstam, L. G., & Greene, C. H. (1999a). Acoustic characterization of *Mysis relicta*. *Limnology and Oceanography, 44*(2), 371-381. doi:10.4319/lo.1999.44.2.0371

Gal, G., Rudstam, L. G., Mills, E. L., Lantry, J. R., Johannsson, O. E., & Greene, C. H. (2006). Mysid and fish zooplanktivory in Lake Ontario: quantification of direct and indirect effects. *Canadian Journal of Fisheries and Aquatic Sciences, 63*(12), 2734-2747. doi:10.1139/F06-156

Goldman, C. R., Morgan, M. D., Threlkeld, S. T., & Angeli, N. (1979). A population dynamics analysis of the cladoceran disappearance from Lake Tahoe, California-Nevada. *Limnology and Oceanography, 24*(2), 289-297. doi:10.4319/lo.1979.24.2.0289

Gorokhova, E. (1998). Exploring and modeling the growth dynamics of *Mysis mixta*. *Ecological Modelling, 110*(1), 45-54. doi:Doi 10.1016/S0304-3800(98)00040-4

Gorokhova, E. (2002). Moult cycle and its chronology in *Mysis mixta* and *Neomysis integer* (Crustacea, Mysidacea): implications for growth assessment. *Journal of Experimental Marine Biology and Ecology, 278*(2), 179-194. doi:10.1016/S0022-0981(02)00333-7

Gorokhova, E. (2009). Toxic cyanobacteria *Nodularia spumigena* in the diet of Baltic mysids: Evidence from molecular diet analysis. *Harmful Algae, 8*(2), 264-272. doi:10.1016/j.hal.2008.06.006

Gorokhova, E., & Hansson, S. (1997). Effects of experimental conditions on the feeding rate of *Mysis mixta* (Crustacea, Mysidacea). *Hydrobiologia, 355*, 167-172. doi:10.1023/A:1003051307725

Gorokhova, E., & Hansson, S. (1999). An experimental study on variations in stable carbon and nitrogen isotope fractionation during growth of *Mysis mixta* and *Neomysis integer*. *Canadian Journal of Fisheries and Aquatic Sciences, 56*(11), 2203-2210. doi:10.1139/cjfas-56-11-2203

Gorokhova, E., & Hansson, S. (2000). Elemental composition of *Mysis mixta* (Crustacea, Mysidacea) and energy costs of reproduction and embryogenesis under laboratory conditions. *Journal of Experimental Marine Biology and Ecology, 246*(1), 103-123. doi:10.1016/S0022-0981(99)00173-2

Gorokhova, E., & Lehtiniemi, M. (2007). A combined approach to understand trophic interactions between *Cercopagis pengoi* (Cladocera: Onychopoda) and mysids in the Gulf of Finland. *Limnology and Oceanography, 52*(2), 685-695. doi:10.4319/lo.2007.52.2.0685

Green, R. H. (1965). *The population ecology of the glacial relict amphipod Pontoporeia affinis Lindstrom in Cayuga Lake, New York.* (PhD), Cornell University, Ithaca, NY, USA.

Gregg, R. E. (1976). *The ecology of Mysis relicta in Twin Lakes, Colorado.* (MSc), Colorado State University, Fort Collins, CO, USA.

Griffiths, D. (2007). Effects of climatic change and eutrophication on the glacial relict, *Mysis relicta*, in Lough Neagh. *Freshwater Biology, 52*(10), 1957-1967. doi:10.1111/j.1365-2427.2007.01824.x

Griffiths, D., Macintosh, K. A., Forasacco, E., Rippey, B., Vaughan, L., McElarney, Y. R., & Gallagher, K. (2015). *Mysis salemaai* in Ireland: new occurrences and existing population declines. *Biology and Environment-Proceedings of the Royal Irish Academy, 115b*(1), 59-65. doi:10.3318/Bioe.2015.06

Grossnickle, N. E. (1978). *The herbivorous and predaceous habits of Mysis relicta in Lake Michigan.* (PhD), University of Wisconsin, Madison, WI, USA.

Grossnickle, N. E. (1982). Feeding habits of *Mysis relicta* - an overview. *Hydrobiologia, 93*(1-2), 101-107. doi:10.1007/Bf00008103

Grossnickle, N. E., & Morgan, M. D. (1979). Density estimates of *Mysis relicta* in Lake Michigan. *Journal of the Fisheries Research Board of Canada, 36*(6), 694-698. doi: 10.1139/f79-103

Gutowski, T. B. (1978). *The ecology of Mysis relicta (Lovén) in Lake Champlain.* (MSc), University of Vermont, Burlington, VT, USA.

Hakala, I. (1978). Distribution, population dynamics and production of *Mysis relicta* (Lovén) in southern Finland. *Annales Zoologici Fennici, 15*(3), 243-258.

Hamilton, A. L. (1971). Zoobenthos of fifteen lakes in the experimental lakes area, Northwestern Ontario. *Journal of the Fisheries Research Board of Canada, 28*(2), 257-263. doi:10.1139/f71-036

Hamrén, U., & Hansson, S. (1999). A mysid shrimp (*Mysis mixta*) is able to detect the odour of its predator (*Clupea harengus*). *Ophelia, 51*(3), 187-191. doi:10.1080/00785326.1999.10409408

Hansson, S., De Stasio, B. T., Gorokhova, E., & Mohammadian, M. A. (2001). Ratio-dependent functional responses - tests with the zooplanktivore *Mysis mixta*. *Marine Ecology Progress Series, 216*, 181-189. doi:DOI 10.3354/meps216181

Hansson, S., Larsson, U., & Johansson, S. (1990). Selective predation by herring and mysids, and zooplankton community structure in a Baltic Sea coastal area. *Journal of Plankton Research, 12*(5), 1099-1116. doi:10.1093/plankt/12.5.1099

Hesthagen, I. H. (1973). Diurnal and seasonal variations in the near-bottom fauna - the hyperbenthos - in one of the deeper channels of the Kieler Bucht (Western Baltic). *Kieler Meeresforsch 29*, 116-140.

Holda, T. J., Rudstam, L. G., Bowen, K. L., Weidel, B. C., Watkins, J. M., Sullivan, P. J., . . . Connerton, M. J. (2019). Status of *Mysis diluviana* in Lake Ontario in 2013: Lower abundance but higher fecundity than in the 1990s. *Journal of Great Lakes Research, 45*(2), 307-316. doi:10.1016/j.jglr.2019.01.007

Holmquist, C. (1963). Some notes on *Mysis relicta* and its relatives in northern Alaska. *Arctic, 16*, 109-128.

Horppila, J., Liljendahl-Nurminen, A., Malinen, T., Salonen, M., Tuomaala, A., Uusitalo, L., & Vinni, M. (2003). *Mysis relicta* in a eutrophic lake: Consequences of obligatory habitat shifts. *Limnology and Oceanography, 48*(3), 1214-1222. doi:10.4319/lo.2003.48.3.1214

Houghton, C. J., Bronte, C. R., Paddock, R. W., & Janssen, J. (2010). Evidence for allochthonous prey delivery to Lake Michigan's Mid-Lake Reef Complex: Are deep reefs analogs to oceanic sea mounts? *Journal of Great Lakes Research, 36*(4), 666-673. doi:10.1016/j.jglr.2010.07.003

Hrycik, A. R., Simonin, P. W., Rudstam, L. G., Parrish, D. L., Pientka, B., & Mihuc, T. B. (2015). *Mysis* zooplanktivory in Lake Champlain: A bioenergetics analysis. *Journal of Great Lakes Research, 41*(2), 492-501. doi:10.1016/j.jglr.2015.03.011

Hyatt, K. D., McQueen, D. J., & Ogden, A. D. (2018). Have invasive mysids (*Mysis diluviana*) altered the capacity of Osoyoos Lake, British Columbia to produce sockeye salmon (*Oncorhynchus nerka*). *The Open Fish Science Journal 11*, 1-26. doi:10.2174/1874401X01811010001

Iacarella, J. C., Dick, J. T. A., Alexander, M. E., & Ricciardi, A. (2015). Ecological impacts of invasive alien species along temperature gradients: testing the role of environmental matching. *Ecological Applications, 25*(3), 706-716. doi:10.1890/14-0545.1

Isaac, E. J., Hrabik, T. R., Stockwell, J. D., & Gamble, A. E. (2012). Prey selection by the Lake Superior fish community. *Journal of Great Lakes Research, 38*(2), 326-335. doi:10.1016/j.jglr.2012.02.017

Jensen, O. P., Yurista, P. M., Hrabik, T. R., & Stockwell, J. D. (2009). Densities and diel vertical migration of *Mysis relicta* in Lake Superior: a comparison of optical plankton counter and net-based approaches. *Verhandlungen des Internationalen Verein Limnologie, 30*(6), 957-963. doi:10.1080/03680770.2009.11902279

Johannsson, O. E. (1992). Life history and productivity of *Mysis relicta* in Lake Ontario. *Journal of Great Lakes Research, 18*(1), 154-168. doi:10.1016/S0380-1330(92)71282-9

Johannsson, O. E. (1995). Response of *Mysis relicta* population dynamics and productivity to spatial and seasonal gradients in Lake Ontario. *Canadian Journal of Fisheries and Aquatic Sciences, 52*(7), 1509-1522. doi:10.1139/f95-145

Johannsson, O. E., Bowen, K. L., Holeck, K. T., & Walsh, M. G. (2011). *Mysis diluviana* population and cohort dynamics in Lake Ontario before and after the establishment of *Dreissena* spp., *Cercopagis pengoi*, and *Bythotrephes longimanus*. *Canadian Journal of Fisheries and Aquatic Sciences, 68*(5), 795-811. doi:10.1139/F2011-028

Johannsson, O. E., Bowen, K. L., Wood, C. M., Smith, R. W., Chu, C., Rudstam, L. G., & Boscarino, B. (2008). Relating nucleic acid and protein indices to growth in *Mysis relicta*: ration, cycling temperature, and metabolism. *Aquatic Biology, 4*(1), 33-46. doi:10.3354/ab00091

Johannsson, O. E., Leggett, M. F., Rudstam, L. G., Servos, M. R., Mohammadian, M. A., Gal, G., . . . Hesslein, R. H. (2001). Diet of *Mysis relicta* in Lake Ontario as revealed by stable isotope and gut content analysis. *Canadian Journal of Fisheries and Aquatic Sciences, 58*(10), 1975-1986. doi:10.1139/cjfas-58-10-1975

Johnson, B. M., & Martinez, P. J. (2012). Hydroclimate mediates effects of a keystone species in a coldwater reservoir. *Lake and Reservoir Management, 28*(1), 70-83. doi:10.1080/07438141.2012.658492

Johnson, B. M., Pate, W. M., Silver, D. B., & Sharp, J. L. (2018). Invasion success and population characteristics of the opossum shrimp, *Mysis diluviana*, in Wyoming, USA. *Aquatic Invasions, 13*(3), 409-420. doi:10.3391/ai.2018.13.3.08

Jokela, A., Arnott, S. E., & Beisner, B. E. (2011). Patterns of *Bythotrephes longimanus* distribution relative to native macroinvertebrates and zooplankton prey. *Biological Invasions, 13*(11), 2573-2594. doi:10.1007/s10530-011-0072-1

Juday, C., & Birge, E. A. (1927). *Pontoporeia* and *Mysis* in Wisconsin Lakes. *Ecology, 8*(4), 445-452. doi:10.2307/1930152

Jude, D. J., Rudstam, L. G., Holda, T. J., Watkins, J. M., Euclide, P. T., & Balcer, M. D. (2018). Trends in *Mysis diluviana* abundance in the Great Lakes, 2006-2016. *Journal of Great Lakes Research, 44*(4), 590-599. doi:10.1016/j.jglr.2018.04.006

Kane, W. F. d. V. (1904). Further Captures of Mysis Relicta in Ireland. *The Irish Naturalist, 13*(5), 107-109.

Karlson, A. M. L., & Viitasalo-Frosen, S. (2009). Assimilation of C-14-labelled zooplankton benthic eggs by macrobenthos. *Journal of Plankton Research, 31*(4), 459-463. doi:10.1093/plankt/fbn131

Kelly, J. R., Yurista, P. M., Miller, S. E., Cotter, A. C., Corry, T. C., Scharold, J. V., . . . Stockwell, J. D. (2011). Challenges to Lake Superior's condition, assessment, and management: A few observations across a generation of change. *Aquatic Ecosystem Health & Management, 14*(4), 332-344. doi:10.1080/14634988.2011.626753

Kinsten, B., & Olsén, P. (1981). Impact of *Mysis relicta* Lovén introduction on the plankton of two mountain lakes, Sweden. *Report of the Institute of Freshwater Research, Drottningholm, 59*, 64-74.

Kjellberg, G., Hessen, D. O., & Nilssen, J. P. (1991). Life history, growth and production of *Mysis relicta* in the large, fjord-type Lake Mjosa, Norway. *Freshwater Biology, 26*(2), 165-173. doi:10.1111/j.1365-2427.1991.tb01726.x

Klump, J. V., Kaster, J. L., & Sierszen, M. E. (1991). *Mysis relicta* assimilation of hexachlorobiphenyl from sediments. *Canadian Journal of Fisheries and Aquatic Sciences, 48*(2), 284-289. doi:10.1139/f91-039

Köhn, J., & Waterstraat, A. (1990). Recent distribution of glacial relict Malacostraca in the lakes of Mecklenburg. *Annales Zoologici Fennici, 27*, 237-240.

Koksvik, J. I., Reinertsen, H., & Koksvik, J. (2009). Plankton development in Lake Jonsvatn, Norway, after introduction of *Mysis relicta*: a long-term study. *Aquatic Biology, 5*(3), 293-304. doi:10.3354/ab00158

Kopache, M. E., & Siegried, C. A. (1982). Notes on the ecology of *Mysis relicta* in Lake George. In *The Lake George Ecosystem: A Compilation* (Vol. II, pp. 49-61).

Landrum, P. F., Frez, W. A., & Simmons, M. S. (1992). The effect of food-consumption on the toxicokinetics of benzo(a)pyrene and 2,2',4,4',5,5'-hexachlorobiphenyl in *Mysis relicta*. *Chemosphere, 25*(3), 397-415. doi:10.1016/0045-6535(92)90556-7

Langeland, A. (1981). Decreased zooplankton density in two Norwegian lakes caused by predation of recently introduced *Mysis relicta*. *Verhandlungen des Internationalen Verein Limnologie, 21*(2), 926-937. doi:10.1080/03680770.1980.11897112

Langeland, A. (1988). Decreased zooplankton density in a mountain lake resulting from predation by recently introduced *Mysis relicta*. *Verhandlungen des Internationalen Verein Limnologie, 23*(1), 419-429. doi:10.1080/03680770.1987.11897956

Langeland, A., Carl, L. M., Hicks, F. J., & Monroe, B. (1991a). Impact of predation by *Mysis relicta* and fish on zooplankton in four oligotrophic, north temperate lakes. *American Fisheries Society Symposium, 9*, 88-97.

Langeland, A., Koksvik, J. I., & Nydal, J. (1991b). Impact of the introduction of *Mysis relicta* on the zooplankton and fish populations in a Norwegian lake. *American Fisheries Society Symposium, 9*, 98-114.

Larkin, P. A. (1948). *Pontoporeia* and *Mysis* in Athabaska, Great Bear and Great Slave Lakes. *Bulletin of the Fisheries Research Board of Canada, 78*, 1-33.

Lasenby, D. C. (1971). *The ecology of Mysis relicta in an Arctic and a temperate lake.* (PhD), University of Toronto, Toronto, ON, CA.

Lasenby, D. C., & Fürst, M. (1981). Feeding of *Mysis relicta* Lovén on macrozooplankton. *Report of the Institute of Freshwater Research, Drottningholm, 59*, 75-80.

Lasenby, D. C., & Langford, R. R. (1972). Growth, life history, and respiration of *Mysis relicta* in an Arctic and temperate lake. *Journal of the Fisheries Research Board of Canada, 29*(12), 1701-1708. doi:10.1139/f72-270

Lasenby, D. C., & Langford, R. R. (1973). Feeding and assimilation of *Mysis relicta*. *Limnology and Oceanography, 18*(2), 280-285. doi:10.4319/lo.1973.18.2.0280

Lasenby, D. C., & Sherman, R. K. (1991). Design and evaluation of a bottom-closing net used to capture mysids and other suprabenthic fauna. *Canadian Journal of Zoology, 69*(3), 783-786. doi:10.1139/z91-113

Lasenby, D. C., & Shi, Y. Q. (2004). Changes in the elemental composition of the stomach contents of the opossum shrimp *Mysis relicta* during diel vertical migration. *Canadian Journal of Zoology, 82*(3), 525-528. doi:10.1139/Z04-005

Lehman, J. T., Bowers, J. A., Gensemer, R. W., Warren, G. J., & Branstrator, D. K. (1990). *Mysis relicta* in Lake Michigan - abundances and relationships with their potential prey, *Daphnia*. *Canadian Journal of Fisheries and Aquatic Sciences, 47*(5), 977-983. doi:10.1139/f90-112

Lehtiniemi, M., Kiljunen, M., & Jones, R. I. (2009). Winter food utilisation by sympatric mysids in the Baltic Sea, studied by combined gut content and stable isotope analyses. *Marine Biology, 156*(4), 619-628. doi:10.1007/s00227-008-1113-x

Lehtiniemi, M., & Lindén, E. (2006). *Cercopagis pengoi* and *Mysis* spp. alter their feeding rate and prey selection under predation risk of herring (*Clupea harengus membras*). *Marine Biology, 149*(4), 845-854. doi:10.1007/s00227-006-0243-2

Lehtiniemi, M., Viitasalo, M., & Kuosa, H. (2002). Diet composition influences the growth of the pelagic mysid shrimp, *Mysis mixta* (Mysidacea). *Boreal Environment Research, 7*(2), 121-128.

Lester, D. C., & McIntosh, A. (1994). Accumulation of polychlorinated biphenyl congeners from Lake Champlain sediments by *Mysis relicta*. *Environmental Toxicology and Chemistry, 13*(11), 1825-1841. doi:10.1002/etc.5620131115

Levy, D. A. (1991). Acoustic analysis of diel vertical migration behavior of *Mysis relicta* and kokanee (*Oncorhynchus nerka*) within Okanagan Lake, British Columbia. *Canadian Journal of Fisheries and Aquatic Sciences, 48*(1), 67-72. doi:10.1139/f91-010

Liljendahl-Nurminen, A., Horppila, J., Uusitalo, L., & Niemistö, J. (2008). Spatial variability in the abundance of pelagic invertebrate predators in relation to depth and turbidity. *Aquatic Ecology, 42*(1), 25-33. doi:10.1007/s10452-006-9070-2

Lindén, E., & Kuosa, H. (2004). Effects of grazing and excretion by pelagic mysids (*Mysis* spp.) on the, size structure and biomass of the phytoplankton community. *Hydrobiologia, 514*, 73-78. doi:10.1007/978-94-017-0920-0_6

MacNeil, C., & Dick, J. T. A. (2012). Differential predatory and interference interactions between native and invasive freshwater amphipods and a co-occurring mysid (Crustacea). *Hydrobiologia, 683*(1), 35-42. doi:10.1007/s10750-011-0938-3

Madeira, P. T., Brooks, A. S., & Seale, D. B. (1982). Excretion of total phosphorus, dissolved reactive phosphorus, ammonia, and urea by Lake Michigan *Mysis relicta*. In M. D. Morgan (Ed.), *Ecology of Mysidacea* (pp. 145-154). Dordrecht, Netherlands: Springer.

Margoński, P., & Maciejewska, K. (1999). The distribution, abundance and biomass of *Mysis mixta* and *Neomysis integer* (Crustacea: Mysidacea) in the open waters of the southern Baltic Sea. *Bulletin of the Sea Fisheries Institute, 147*, 23-35.

Maxwell, S. J. (2003). *Distribution of Mysis diluviana in nearshore Lake Ontario.* (MSc), University of New Hampshire, Durham, NH, USA.

McCoy, A. K. (2015). *An assessment of the impact of non-native lake trout Salvelinus namaycush and Mysis diluviana on the growth and survival of pelagic planktivores in Lake Tahoe.* (PhD), University of Washington, Seattle, WA, USA.

McDonald, M. E., Crowder, L. B., & Brandt, S. B. (1990). Changes in *Mysis* and *Pontoporeia* populations in southeastern Lake Michigan: A response to shifts in the fish community. *Limnology and Oceanography, 35*(1), 220-227. doi:10.4319/lo.1990.35.1.0220

McWilliam, P. S. (1970). *Seasonal changes in abundance and reproduction in the “opossum shrimp”, Mysis relicta Lovén in Lake Michigan.* (MSc), University of Sydney, Sydney, NSW, Australia.

Miller, G. S. (2003). *Mysis* vertical migration in Grand Traverse Bay, Lake Michigan, observed by an acoustic doppler current profiler. *Journal of Great Lakes Research, 29*(3), 427-435. doi:10.1016/S0380-1330(03)70448-1

Moen, V., & Langeland, A. (1989). Diurnal vertical and seasonal horizontal distribution patterns of *Mysis relicta* in a large Norwegian lake. *Journal of Plankton Research, 11*(4), 729-745. doi:10.1093/plankt/11.4.729

Mohammadian, M. A., Hansson, S., & DeStasio, B. T. (1997). Are marine planktonic invertebrates food limited? The functional response of *Mysis mixta* (Crustacea, Mysidacea) in the Baltic Sea. *Marine Ecology Progress Series, 150*(1-3), 113-119. doi:10.3354/meps150113

Morgan, M. D. (1976). *Life history and annual net secondary productivity of Mysis relicta (Lovén) in west central Lake Michigan.* (MSc), University of Wisconsin - Milwaukee, Milwaukee, WI, USA.

Morgan, M. D. (1980). Life history characteristics of two introduced populations of *Mysis relicta*. *Ecology, 61*(3), 551-561. doi:10.2307/1937421

Morgan, M. D. (1981). Abundance, life history, and growth of introduced populations of the opossum shrimp (*Mysis relict*a) in subalpine California Lakes. *Canadian Journal of Fisheries and Aquatic Sciences, 38*(8), 989-993. doi:10.1139/f81-134

Morgan, M. D. (1985). Growth and its relationship to reproduction in *Mysis relicta*. In A. M. Wenner (Ed.), *Factors in adult growth* (pp. 235-250). Boston, MA, USA: A.A. Balkema.

Morgan, M. D., & Beeton, A. M. (1978). Life history and abundance of *Mysis relicta* in Lake Michigan. *Journal of the Fisheries Research Board of Canada, 35*(9), 1165-1170. doi:10.1139/f78-187

Morgan, M. D., & Threlkeld, S. T. (1982). Size dependent horizontal migration of *Mysis relicta*. *Hydrobiologia, 93*(1-2), 63-68. doi:10.1007/Bf00008099

Morgan, M. D., Threlkeld, S. T., & Goldman, C. R. (1978). Impact of the introduction of kokanee (*Oncorhynchus nerka*) and opossum shrimp (*Mysis relicta*) on a subalpine lake. *Journal of the Fisheries Research Board of Canada, 35*, 1572-1579. doi:10.1139/f78-247

Mundie, J. H. (1959). The diurnal activity of the larger invertebrates at the surface of Lac La Ronge, Saskatchewan. *Canadian Journal of Zoology, 37*(6), 945-956. doi:10.1139/z59-090

Naesje, T. F., Jensen, A. J., Moen, V., & Saksgård, R. (1991). Habitat use by zooplankton, *Mysis relicta*, and Arctic char in Lake Jonsvatn, Norway. *American Fisheries Society Symposium, 9*, 75-87.

Naesje, T. F., Saksgard, R., Jensen, A. J., & Sandlund, O. T. (2003). Life history, habitat utilisation, and biomass of introduces *Mysis relicta*. *Limnologica, 33*(4), 244-257. doi:10.1016/S0075-9511(03)80020-8

Nero, R. W., & Davies, I. J. (1982). Comparison of two sampling methods for estimating the abundance and distribution of *Mysis relicta*. *Canadian Journal of Fisheries and Aquatic Sciences, 39*(2), 349-355. doi:10.1139/f82-048

Nero, R. W., & Schindler, D. W. (1983). Decline of *Mysis relicta* during the acidification of Lake 223. *Canadian Journal of Fisheries and Aquatic Sciences, 40*(11), 1905-1911. doi:10.1139/f83-221

Nero, R. W., & Sprules, W. G. (1986). Zooplankton species abundance and biomass in relation to occurrence of *Mysis relicta* (Malacostraca: Mysidacea). *Canadian Journal of Fisheries and Aquatic Sciences, 43*(2), 420-434. doi:10.1139/f86-053

Nordin, L. J., Arts, M. T., Johannsson, O. E., & Taylor, W. D. (2008). An evaluation of the diet of *Mysis relicta* using gut contents and fatty acid profiles in lakes with and without the invader *Bythotrephes longimanus* (Onychopoda, Cercopagidae). *Aquatic Ecology, 42*(3), 421-436. doi:10.1007/s10452-007-9098-y

Nowicki, C. J., Bunnell, D. B., Armenio, P. M., Warner, D. M., Vanderploeg, H. A., Cavaletto, J. F., . . . Adams, J. V. (2017). Biotic and abiotic factors influencing zooplankton vertical distribution in Lake Huron. *Journal of Great Lakes Research, 43*(6), 1044-1054. doi:10.1016/j.jglr.2017.08.004

O'Malley, B. P., & Bunnell, D. B. (2014). Diet of *Mysis diluviana* reveals seasonal patterns of omnivory and consumption of invasive species in offshore Lake Michigan. *Journal of Plankton Research, 36*(4), 989-1002. doi:10.1093/plankt/fbu038

O'Malley, B. P., Dillon, R. A., Paddock, R. W., Hansson, S., & Stockwell, J. D. (2018a). An underwater video system to assess abundance and behavior of epibenthic *Mysis*. *Limnology and Oceanography-Methods, 16*(12), 868-880. doi:10.1002/lom3.10289

O'Malley, B. P., Hansson, S., & Stockwell, J. D. (2018b). Evidence for a size-structured explanation of partial diel vertical migration in mysids. *Journal of Plankton Research, 40*(1), 66-76. doi:10.1093/plankt/fbx060

O'Malley, B. P., Rudstam, L. G., Watkins, J. M., Holda, T. J., & Weidel, B. C. (2017). Effects of food web changes on *Mysis diluviana* diet in Lake Ontario. *Journal of Great Lakes Research, 43*(5), 813-822. doi:10.1016/j.jglr.2017.02.003

O'Malley, B. P., & Stockwell, J. D. (2019). Diel feeding behavior in a partially migrant *Mysis* population: A benthic-pelagic comparison. *Food Webs, 20*, e00117. doi:10.1016/j.fooweb.2019.e00117

Ogonowski, M., Duberg, J., Hansson, S., & Gorokhova, E. (2013a). Behavioral, ecological and genetic differentiation in an open environment - a study of a mysid population in the Baltic Sea. *Plos One, 8*(3). doi:10.1371/journal.pone.0057210

Ogonowski, M., Hansson, S., & Duberg, J. (2013b). Status and vertical size-distributions of a pelagic mysid community in the northern Baltic proper. *Boreal Environment Research, 18*(1), 1-18.

Oliver, S. K., Branstrator, D. K., Hrabik, T. R., Guildford, S. J., Hecky, R. E., & Smith, R. (2015). Nutrient excretion by crustacean zooplankton in the deep chlorophyll layer of Lake Superior. *Canadian Journal of Fisheries and Aquatic Sciences, 72*(3), 390-399. doi:10.1139/cjfas-2014-0209

Parker, J. I. (1980). Predation by *Mysis relicta* on *Pontoporeia hoyi*: A food chain link of potential importance in the Great Lakes. *Journal of Great Lakes Research, 6*(2), 164-166. doi:10.1016/s0380-1330(80)72095-6

Paterson, M. J., Podemski, C. L., Wesson, L. J., & Dupuis, A. P. (2011). The effects of an experimental freshwater cage aquaculture operation on *Mysis diluviana*. *Journal of Plankton Research, 33*(1), 25-36. doi:10.1093/plankt/fbq096

Penk, M. R. (2011). A review of the current distribution of the freshwater opossum shrimp *Mysis salemaai* Audzijonyte and Vainola, 2005 in Ireland. *Biology and Environment-Proceedings of the Royal Irish Academy, 111b*(2), 107-115. doi:10.3318/Bioe.2011.09

Penk, M. R., Donohue, I., & Irvine, K. (2018). Temporally variable niche overlap and competitive potential of an introduced and a native mysid shrimp. *Hydrobiologia, 823*(1), 109-119. doi:10.1007/s10750-018-3700-2

Penk, M. R., Donohue, I., Minchin, D., & Irvine, K. (2016). Life history timing, but not body size, of *Mysis salemaai* (Crustacea: Mysida) conserved across a trophic gradient at its southern distribution. *Hydrobiologia, 775*(1), 83-95. doi:10.1007/s10750-016-2716-8

Penk, M. R., & Minchin, D. (2014). Seasonal migration of a glacial relict mysid (Crustacea) into the littoral zone and its co-occurrence with an introduced competitor in Lough Derg (Ireland). *Hydrobiologia, 726*(1), 1-11. doi:10.1007/s10750-013-1744-x

Pothoven, S. A., Fahnenstiel, G. L., & Vanderploeg, H. A. (2004). Spatial distribution, biomass and population dynamics of *Mysis relicta* in Lake Michigan. *Hydrobiologia, 522*(1-3), 291-299. doi:10.1023/B:HYDR.0000029982.52263.c0

Pothoven, S. A., Fahnenstiel, G. L., & Vanderploeg, H. A. (2010). Temporal trends in *Mysis relicta* abundance, production, and life-history characteristics in southeastern Lake Michigan. *Journal of Great Lakes Research, 36*, 60-64. doi:10.1016/j.jglr.2010.03.008

Pothoven, S. A., Fahnenstiel, G. L., Vanderploeg, H. A., & Luttenton, M. (2000). Population dynamics of *Mysis relicta* in southeastern Lake Michigan, 1995-1998. *Journal of Great Lakes Research, 26*(4), 357-365. doi:10.1016/S0380-1330(00)70700-3

Pothoven, S. A., Fanslow, D. L., & Fahnenstiel, G. L. (2012). Lipid content of *Mysis diluviana* in the offshore region of southeastern Lake Michigan in 2009-2010. *Journal of Great Lakes Research, 38*(3), 561-568. doi:10.1016/j.jglr.2012.05.003

Pothoven, S. A., & Vanderploeg, H. A. (2004). Diet and prey selection of alewives in Lake Michigan: Seasonal, depth, and interannual patterns. *Transactions of the American Fisheries Society, 133*(5), 1068-1077. doi:10.1577/T03-110.1

Pothoven, S. A., & Vanderploeg, H. A. (2017). Changes in *Mysis diluviana* abundance and life history patterns following a shift toward oligotrophy in Lake Michigan. *Fundamental and Applied Limnology, 190*(3), 199-212. doi:10.1127/fal/2017/1039

Quirt, J., & Lasenby, D. (2002). Cannibalism and ontogenetic changes in the response of the freshwater shrimp *Mysis relicta* to chemical cues from conspecific predators. *Canadian Journal of Zoology, 80*(6), 1022-1025. doi:10.1139/Z02-084

Ragnarsson-Stabo, H., Vrede, T., Axenrot, T., & Sandstrom, A. (2014). Can multi-frequency acoustics improve the monitoring of large zooplankton in large temperate lakes? *Aquatic Ecosystem Health & Management, 17*(4), 374-381. doi:10.1080/14634988.2014.975097

Ramcharan, C. W., & Sprules, W. G. (1986). Visual predation in *Mysis relicta* Lovén. *Verhandlungen des Internationalen Verein Limnologie, 22*, 3215-3219.

Ramcharan, C. W., Sprules, W. G., & Nero, R. W. (1985). Notes on the tactile feeding behaviour of *Mysis relicta* Lovén (Malacostraca: Mysidacea). *Verhandlungen des Internationalen Verein Limnologie, 22*, 3215-3219.

Rennie, M. D., Kennedy, P. J., Mills, K. H., Rodgers, C. M. C., Charles, C., Hrenchuk, L. E., . . . Podemski, C. L. (2019). Impacts of freshwater aquaculture on fish communities: A whole-ecosystem experimental approach. *Freshwater Biology, 64*(5), 870-885. doi:10.1111/fwb.13269

Reynolds, J. B., & DeGraeve, G. M. (1972). Seasonal population characteristics of the opossum shrimp, *Mysis relicta*, in southeastern Lake Michigan, 1970-71. *Proceedings of the 15th Conference on Great Lakes Research, 15*, 117-131.

Richards, R. C., Goldman, C. R., Frantz, T. C., & Wickwire, R. (1975). Where have all the Daphnia gone? The decline of a major cladoceran in Lake Tahoe, California-Nevada. *Verhandlungen des Internationalen Verein Limnologie, 19*(2), 835-842. doi:10.1080/03680770.1974.11896129

Rieman, B. E., & Falter, C. M. (1981). Effects of the establishment of *Mysis relicta* on the macrozooplankton of a large lake. *Transactions of the American Fisheries Society, 110*(5), 613-620. doi:10.1577/1548-8659(1981)110<613:Eoteom>2.0.Co;2

Robertson, A., Powers, C. F., & Anderson, R. F. (1968). Direct observations on *Mysis relicta* from a submarine. *Limnology and Oceanography, 13*(4), 700-702. doi:10.4319/lo.1968.13.4.0700

Rosenberg, R. (1972). Benthic faunal recovery in a Swedish fjord following the closure of a sulphite pulp mill. *Oikos, 23*(1). doi:10.2307/3543930

Rudstam, L. G., Danielsson, K., Hansson, S., & Johansson, S. (1989). Diel vertical migration and feeding patterns of *Mysis mixta* (Crustacea, Mysidacea) in the Baltic Sea. *Marine Biology, 101*(1), 43-52. doi:10.1007/Bf00393476

Rudstam, L. G., Hansson, S., & Larsson, U. (1986). Abundance, species composition and production of mysid shrimps in a coastal area of the northern Baltic proper. *Ophelia, Suppl. 4*, 225-238.

Rudstam, L. G., Hetherington, A. L., & Mohammadian, A. M. (1999). Effect of temperature on feeding and survival of *Mysis relicta*. *Journal of Great Lakes Research, 25*(2), 363-371.

Rudstam, L. G., Knudsen, F. R., Balk, H., Gal, G., Boscarino, B. T., & Axenrot, T. (2008a). Acoustic characterization of *Mysis relicta* at multiple frequencies. *Canadian Journal of Fisheries and Aquatic Sciences, 65*(12), 2769-2779. doi:10.1139/F08-179

Rudstam, L. G., Schaner, T., Gal, G., Boscarino, B., O'Gorman, R., Warner, D., . . . Bowen, K. (2008b). Hydroacoustic measures of *Mysis relicta* abundance and distribution in Lake Ontario. *Aquatic Ecosystem Health & Management, 11*(4), 355-367. doi:10.1080/14634980802539708

Rybock, J. T. (1978). *Mysis relicta Lovén in Lake Tahoe: vertical distribution and nocturnal predation.* (PhD), University of California, Davis, Davis, CA, USA.

Salemaa, H., Tyystjarvimuuronen, K., & Aro, E. (1986). Life histories, distribution and abundance of *Mysis mixta* and *Mysis relicta* in the Northern Baltic Sea. *Ophelia, Suppl. 4*, 239-247.

Salemaa, H., Vuorinen, I., & Välipakka, P. (1990). The distribution and abundance of *Mysis* populations in the Baltic Sea. *Annales Zoologici Fennici, 27*(3), 253-257.

Särkkä, J. (1972). The bottom macrofauna of the oligotrophic Lake Konnevesi, Finland. *Annales Zoologici Fennici, 9*, 141-146.

Scharf, J., & Koschel, R. (2004). Distribution, abundance and life history of *Mysis relicta* (Lovén) in the Feldberg Lake District, Germany. *Limnologica, 34*(3), 199-212. doi:10.1016/S0075-9511(04)80045-8

Scharf, J., & Koschel, R. (2005). Food composition and selectivity of *Mysis relicta* (Lovén) in Lake Breiter Luzin. *Verhandlungen des Internationalen Verein Limnologie, 29*, 208-211.

Scharf, J., Krappe, M., Koschel, R., & Waterstraat, A. (2008). Feeding of European cisco (*Coregonus albula* and *C. lucinensis*) on the glacial relict crustacean *Mysis relicta* in Lake Breiter Luzin (Germany). *Limnologica, 38*(2), 147-158. doi:10.1016/j.limno.2007.12.001

Schindler, D. E., Carter, J. L., Francis, T. B., Lisi, P. J., Askey, P. J., & Sebastian, D. C. (2012). *Mysis* in the Okanagan Lake food web: a time-series analysis of interaction strengths in an invaded plankton community. *Aquatic Ecology, 46*(2), 215-227. doi:10.1007/s10452-012-9393-0

Schoen, E. R., Beauchamp, D. A., Buettner, A. R., & Overman, N. C. (2015). Temperature and depth mediate resource competition and apparent competition between *Mysis diluviana* and kokanee. *Ecological Applications, 25*(7), 1962-1975. doi:10.1890/14-1822.1

Seckar, D. (2009). *Interactions between Chaoborus spp. and Mysis relicta and their impact on pelagic crustacean zooplankton in mesocosms at the Experimental Lakes Area.* (MSc), University of Manitoba, Winnipeg, MB, CA.

Sell, D. W. (1982). Size-frequency estimates of secondary production by *Mysis relicta* in Lakes Michigan and Huron. *Hydrobiologia, 93*(1-2), 69-78. doi:10.1007/Bf00008100

Sellers, T. J. (1995). *The distribution of lake trout, Salvelinus namaycush, and opossum shrimp, Mysis relicta, in small boreal lakes wieth respect to temperature, dissolved oxygen, and light.* (MSc), University of Alberta, Edmonton, AB, CA.

Shea, M. A., & Makarewicz, J. C. (1989). Production, biomass, and trophic interactions of *Mysis relicta* in Lake Ontario. *Journal of Great Lakes Research, 15*(2), 223-232. doi:10.1016/S0380-1330(89)71478-7

Sierszen, M. E., Kelly, J. R., Corry, T. D., Scharold, J. V., & Yurista, P. M. (2011). Benthic and pelagic contributions to *Mysis* nutrition across Lake Superior. *Canadian Journal of Fisheries and Aquatic Sciences, 68*(6), 1051-1063. doi:10.1139/F2011-033

Silver, D. B., Johnson, B. M., Pate, W. M., Christianson, K. R., Tipton, J., Sherwood, J., . . . Hao, Y. (2016). Effect of net size on estimates of abundance, size, age and sex ratio of *Mysis diluviana*. *Journal of Great Lakes Research, 42*(3), 731-737. doi:10.1016/j.jglr.2016.02.012

Simm, M., & Kotta, I. (1992). The abundance and distribution of *Mysis* in the Gulf of Finland. In J. Köhn, M. B. Jones, & A. Moffat (Eds.), *Taxonomy, Biology, and Ecology of (Baltic) Mysids* (pp. 55-60): Rostock University.

Slife, C. (2017). *Life-cycle durations and bioaccumulation of polychlorinated biphenyls by Mysis diluviana in the Finger Lakes of New York.* (MSc), SUNY-ESF, Syracuse, NY, USA.

Smokorowski, K. E. (1998). *The response of the freshwater shrimp, Mysis relicta, to the partial fertilization of Kootenay Lake, British Columbia.* (PhD), Trent University, Peterborough, ON, CA.

Southern, R., & Gardiner, A. C. (1932). Reports from the Limnological Laboratory. II. The diurnal migrations of the crustacea of the plankton in Lough Derg. *Proceedings of the Royal Irish Academy. Section B Biological, Geological, and Chemical Science, 40*(1932/1932), 121-159.

Spencer, C. N., Potter, D. S., Bukantis, R. T., & Stanford, J. A. (1999). Impact of predation by *Mysis relicta* on zooplankton in Flathead Lake, Montana, USA. *Journal of Plankton Research, 21*(1), 51-64. doi:10.1093/plankt/21.1.51

Sullivan, P. J., & Rudstam, L. G. (2016). Quantifying acoustic survey uncertainty using Bayesian hierarchical modeling with an application to assessing *Mysis relicta* population densities in Lake Ontario. *Ices Journal of Marine Science, 73*(8), 2104-2111. doi:10.1093/icesjms/fsw080

Threlkeld, S. T., Rybock, J. T., Morgan, M. D., Folt, C. L., & Goldman, C. R. (1980). The effects of an introduced invertebrate predator and food resource variation on zooplankton dynamics in an ultraoligotrophic lake. In W. C. Kerfoot (Ed.), *Evolution and Ecology of Zooplankton Communities*. Hanover, NH, USA: University Press of New England.

Vainola, R., & Vainio, J. K. (1998). Distributions, life cycles and hybridization of two *Mysis relicta* group species (Crustacea : Mysida) in the northern Baltic Sea and Lake Baven. *Hydrobiologia, 368*, 137-148. doi:10.1023/A:1003237829779

Van Duyn-Henderson, J. A., & Lasenby, D. C. (1986). Zinc and cadmium transport by the vertically migrating opossum shrimp, *Mysis relicta*. *Canadian Journal of Fisheries and Aquatic Sciences, 43*(9), 1726-1732. doi:10.1139/f86-216

Viherluoto, M., Kuosa, H., Flinkman, J., & Viitasalo, M. (2000). Food utilisation of pelagic mysids, *Mysis mixta* and *M. relicta*, during their growing season in the northern Baltic Sea. *Marine Biology, 136*(3), 553-559. doi:10.1007/s002270050715

Viherluoto, M., & Viitasalo, M. (2001a). Effect of light on the feeding rates of pelagic and littoral mysid shrimps: a trade-off between feeding success and predation avoidance. *Journal of Experimental Marine Biology and Ecology, 261*(2), 237-244. doi:10.1016/s0022-0981(01)00277-5

Viherluoto, M., & Viitasalo, M. (2001b). Temporal variability in functional responses and prey selectivity of the pelagic mysid, *Mysis mixta*, in natural prey assemblages. *Marine Biology, 138*(3), 575-583. doi:10.1007/s002270000478

Viitasalo, S. (2007). Effects of bioturbation by three macrozoobenthic species and predation by necto-benthic mysids on cladoceran benthic eggs. *Marine Ecology Progress Series, 336*, 131-140. doi:10.3354/meps336131

Viitasalo, S., & Viitasalo, M. (2004). Predation by the mysid shrimps *Mysis mixta* and *M. relicta* on benthic eggs of *Bosmina longispina* *maritima* (Cladocera) in the northern Baltic Sea. *Marine Ecology Progress Series, 281*, 155-163. doi:10.3354/meps281155

Wang, Y., Consi, T. R., Hansen, T., & Janssen, J. (2012). The relationship between coastal *Mysis diluviana* abundance and spring thermal bar dynamics. *Journal of Great Lakes Research, 38*, 68-72. doi:10.1016/j.jglr.2011.11.016

Waterstraat, A., Krappe, M., Riel, P., & Rumpf, M. (2005). Habitat shifts of *Mysis relicta* (Decapoda, Mysidacea) in the lakes Breiter and Schmaler Luzin (NE Germany). *Crustaceana, 78*, 685-699. doi:10.1163/156854005774353467

Watkins, J. M., Rudstam, L. G., Connerton, M. J., Schaner, T., Rudstam, P. G., & Bowen, K. L. (2015). Abundance and spatial distribution of *Mysis diluviana* in Lake Ontario in 2008 estimated with 120 kHz hydroacoustic surveys and net tows. *Aquatic Ecosystem Health & Management, 18*(1), 63-75. doi:10.1080/14634988.2014.965646

Whall, J. D., & Lasenby, D. C. (2009). Differences in the trophic role of *Mysis diluviana* in two intermontane lakes. *Aquatic Biology, 5*(3), 281-292. doi:10.3354/ab00162

Zyblut, E. R. (1967). *Temporal and spatial changes in distribution and abundance of macro-zooplankton in a large British Columbia lake.* (MSc), University of British Columbia, Vancouver, BC, CA.
